# Structures of three disubstituted [13]-macro­di­lac­tones reveal effects of substitution on macrocycle conformation

**DOI:** 10.1107/S2056989020012037

**Published:** 2020-09-08

**Authors:** Kelli M. Rutledge, Caleb Griesbach, Brandon Q. Mercado, Mark W. Peczuh

**Affiliations:** a Department of Chemistry, University of Connecticut, 55 N. Eagleville Road, U3060, Storrs, CT 06269, USA; b Department of Chemistry, Yale University, PO Box 208107, New Haven, CT 06520, USA

**Keywords:** macrocycle, conformation, planar chirality, crystal structure

## Abstract

The synthesis and crystal structures of three new disubstituted [13]-macro­di­lactones, namely, *trans*-4,8-dimethyl-1,10-dioxa­cyclo­tridec-5-ene-2,9-dione, C_13_H_20_O_4_, **I**, *cis*-4-(4-bromo­phen­yl)-13-methyl-1,10-dioxa­cyclo­tridec-5-ene-2,9-dione C_18_H_21_BrO_4_, **II**, and *trans*-11-methyl-4-phenyl-1,10-dioxa­cyclo­tridec-5-ene-2,9-dione, C_18_H_22_O_4_, **III**, are reported and their conformations are put in the context of other [13]-macrodilactone structures reported previously. Together, they show that the number, location, and relative disposition of groups attached at the termini of planar units of the [13]-macrodilactones subtly influence their aspect ratios.

## Abstract 2

The synthesis and crystal structures of three new disubstituted [13]-macrodilactones, *trans*-4,8-dimethyl-1,10-dioxa­cyclo­tridec-5-ene-2,9-dione, *cis*-4-(4-bromo­phen­yl)-13-methyl-1,10-dioxa­cyclo­tridec-5-ene-2,9-dione, and *trans*-11-methyl-4-phenyl-1,10-dioxa­cyclo­tridec-5-ene-2,9-dione are reported and their conformations are put in the context of other [13]-macrodilactone structures reported previously, showing that the number, location, and relative disposition of groups attached at the termini of planar units of the [13]-macrodilactones subtly influence their aspect ratios.

## Chemical context   

Macrocyclic rings adopt particular conformations by balancing the contributions of multiple, local domain features. We have studied the synthesis, structure, and function of a specific family of macrocycles, the [13]-macrodilactones. These macrocycles, which are made more rigid by ester and alkene planar units, minimize transannular inter­actions of substit­uents at stereogenic centers along their backbone. The overall effect of the number of atoms in the ring, the planar units, and the stereogenic centers promotes the adoption of a conformation that contains an element of planar chirality.
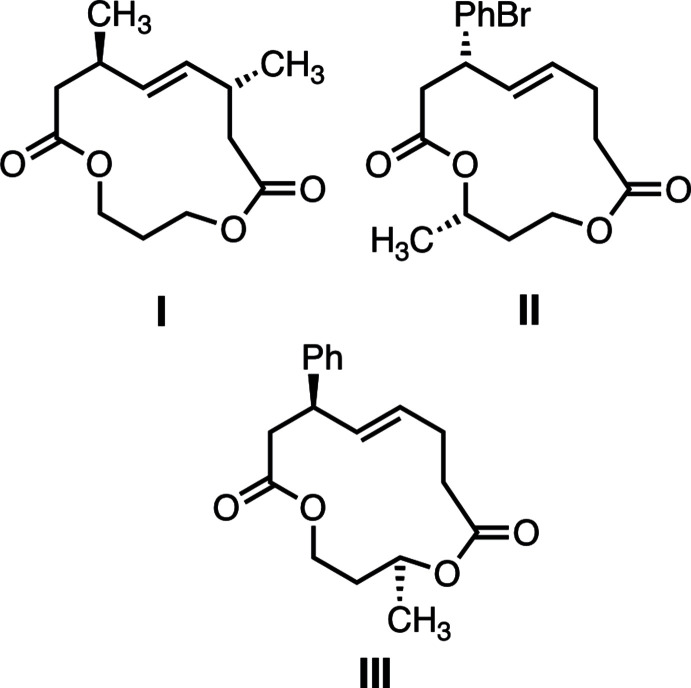


**Figure 1:**
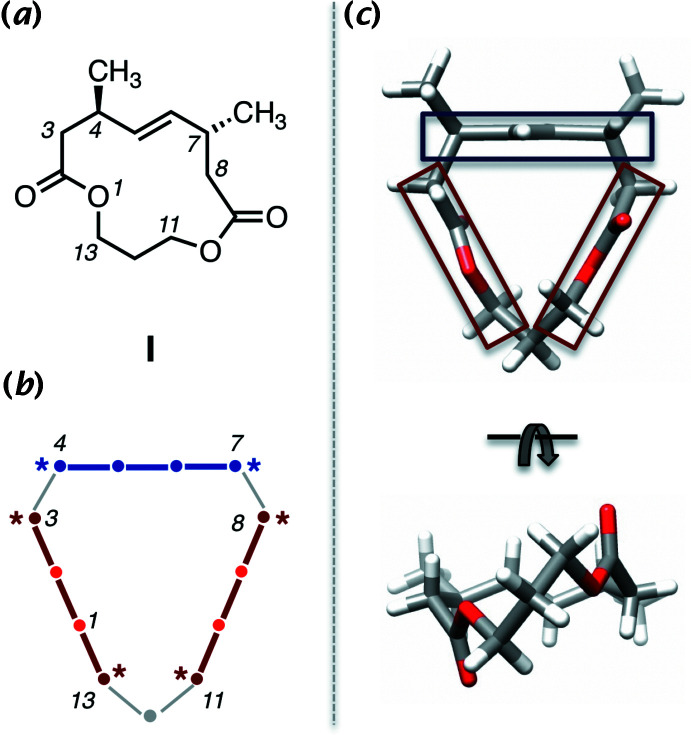
Ribbon motif of [13]-macrodilactones. (*a*) Structure and number of [13]-macrodilactones using compound **I** as an illustration. (*b*) Schematic of the ring showing the three planar units: two esters (brown) and alkene (blue). Key atoms at the termini of the planar units are marked with asterisks. (*c*) Mol­ecular structure of **I** from X-ray data.

The modularity of macrocycles lends to their attractiveness as scaffolds for the development of new bioactive compounds (Whitty *et al.*, 2017 ▸; Yudin, 2015 ▸; Driggers *et al.*, 2008 ▸). Macrocycles have mini-domains of a few atoms that can influence the conformation of the ring, modulate rigidity/flexibility, and tune their physicochemical and biochemical properties (Whitty *et al.*, 2016 ▸; Larsen *et al.*, 2015 ▸). For the [13]-macrodilactone motif exemplified by *trans*-4,8-dimethyl-1,10-dioxa­cyclo­tridec-5-ene-2,9-dione **I** in Fig. 1 ▸, two four-atom ester units (C13/O1/C2/C3 and C8/C9/O10/C11) are linked *via* a central carbon (C12) and one *trans*-2-butenyl moiety (atoms C4–C7). By virtue of the planarity of the multi-atom units, the ring is significantly stiffened compared to a saturated thirteen-membered ring. The conformation, which is informally referred to as the ‘ribbon’ conformation, arises from a balance between the number of atoms that make up the ring and the nature of the planar units that reduce its flexibility. There is a planar chirality associated with the macrocycle (only the *R*-configured plane, *pR*, form is shown for **I**) that arises from the asymmetry around the alkene unit as it orients itself perpendicular to the mean plane of the macrocycle. The planar chirality of the [13]-macrodilactones is directly analogous to that of *E*-cyclo­octene (Fyvie & Peczuh, 2008*a* ▸,*b* ▸; Eliel & Wilen, 1994 ▸). Viewed from above (parallel to the alkene), the ribbon appears roughly triangular (Fig. 1 ▸ *c*, top), with a long axis and a short axis.

Here we report on the synthesis and solid-state structural characterization of [13]-macrodilactones **I**, **II**, and **III**. These new structures, along with eight more previously reported [13]-macrodilactone structures, are analyzed to assess how substitution at specific atoms of the backbone influences the conformation.

## Structural commentary   

**Figure 2:**
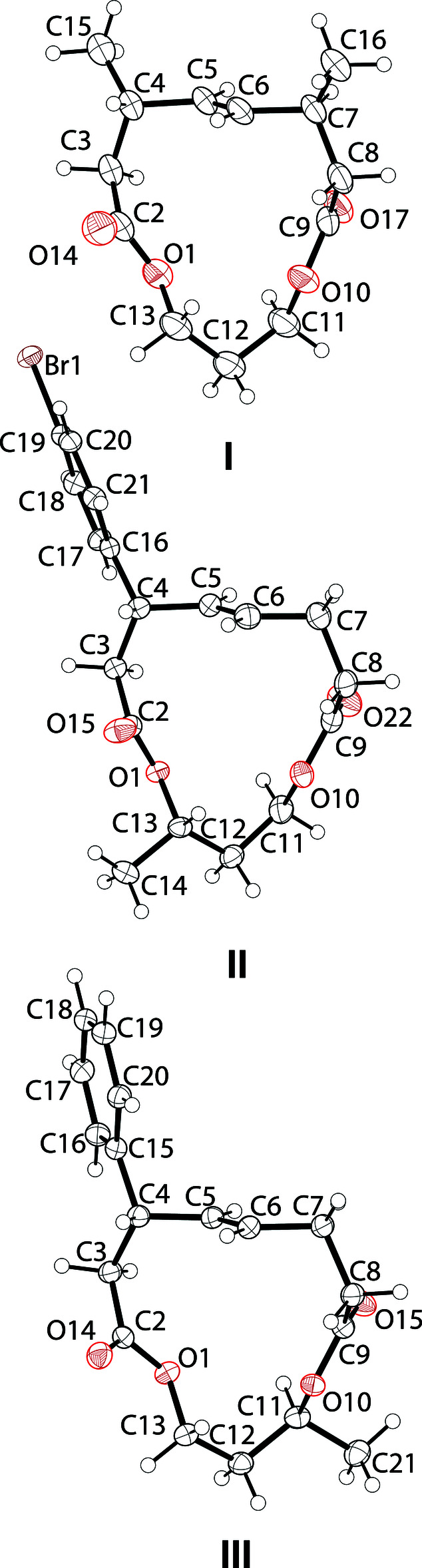
The mol­ecular structures of **I**, **II**, and **III** with 50% displacement ellipsoid probability levels. Note that the structures here are of the *pS-*configured planar chirality.

Each of the new structures has two stereogenic centers. The synthetic routes were not stereoselective, and the products were isolated as racemates. Consequently, each compound crystallized as a racemate. The stereogenic centers of compounds **I**, **II**, and **III** seen in Fig. 2 ▸ establish only the relative stereochemistry observed in the asymmetric unit for each one. The only common feature of **I**, **II**, and **III** is the [13]-macrodilactone core. All bond distances and angles are in the expected ranges and unexceptional. A more in depth analysis of mol­ecular aspect ratios can be found in the *Database survey*.

## Database survey   

**Figure 3:**
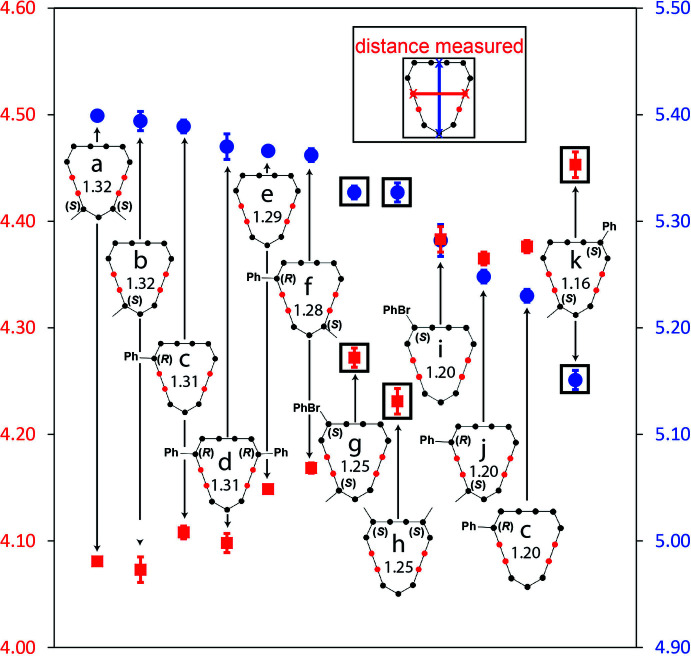
Length, width, and aspect ratios of *pS*-configured [13]-macrodilactones in the ribbon conformation. The inset shows the distances measured in the macrocycles. All error bars are shown to 3σ, but some are smaller than the marker chosen to represent the point. The boxes highlight data from compounds **I** (*h*), **II** (*g*), and **III** (*k*) in this report. All distances are shown in Å.

A survey of the Cambridge Structural Database (CSD) yielded a total of 17 structures of [13]-macrodilactones, counting the three new structures reported here (Table 1 ▸). Of these structures, 11 share the same fundamental ribbon conformation described earlier. Comparison of their structures in light of their substitution patterns along the macrocyclic backbone revealed subtle differences in their conformations. Aspect ratio, defined as the ratio of the C12-to-centroid of C5 and C6 (length, or long-axis) and the C2-to-C9 carbonyl carbon distance (width, or short-axis) of the macrocyclic ring, was our metric to express the changes in conformation. By virtue of the cyclic structure, compression along one axis leads to expansion along the complementary one, and *vice versa*, affecting the aspect. Note that structure *e* (Fig. 3 ▸) is of the unsubstituted [13]-macrodilactone, containing no pendant groups along its backbone; it represents a reference point for comparisons amongst the other substituted macrocycles.

Subtle differences in the aspect ratios of the [13]-macrodilactones depicted in Fig. 3 ▸ were attributed to the location and number but not size of groups attached to the ring, which we found remarkable. For example, the positioning of a single substituted atom affected the aspect ratio as exemplified by *b*, *c*, and *i*. Trends for di-substituted [13]-macrodilactones separated into two groups. In the first group are the ‘symmetrical’ di-substituted compounds: *trans*-11,13- (*a*), *trans* −3,8- (*d*), and *trans*-4,8- (*h*, compound **I**). The trend for this group largely follows that in the monosubstituted series. That is, the aspect ratio increased slightly when substitutions were made on either end of the ester units but decreased upon substitution at the allylic carbons. The second group of di-substituted macrocycles is a catch-all that collects compounds where the substituted carbons are either on the same side of the ring relative to its long-axis [*cis*-4,13- (*g*, compound **II**) and *trans*-3,13 (*j*)] or opposite sides [*cis*-3,11 (*f*) and *trans*-4,11 (*k*, compound **III**)]. These compounds pit substitutions at a site (C11/13) that stretches the long-axis with sites that either also extend (C3/C8) or compress (C4/C7) it. A clear rationale to explain relationships between these substitution patterns and their aspect ratios was not apparent. One observation was that any substitution at the allylic positions tended to compress the aspect ratios of all the [13]-macrodilactones. Aspect ratios ranged from 1.16 on the low end (*q*, compound **III**) to 1.32 (*a*) on the high end. That represents a 14% change in aspect ratio linked only to the number and location of substituted carbons along the backbone of the [13]-macrodilactone structure.

## Computational analysis of conformations   

**Figure 4:**
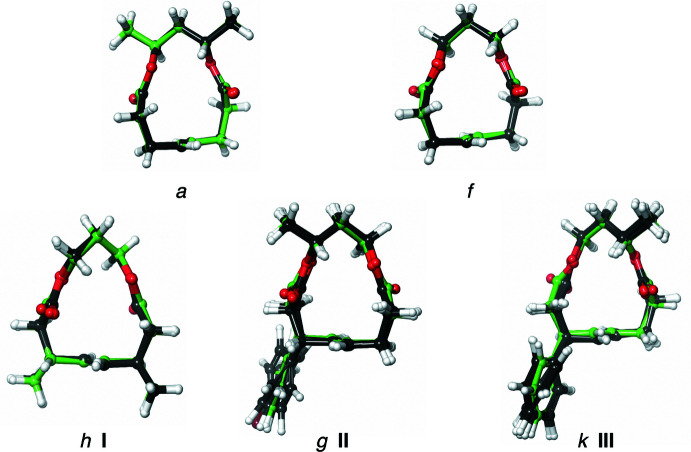
Overlays of crystallographic (green) and the DFT-optimized (black) structures for compounds *a*, *f*, *g* (**II**), *h* (**I**), and *k* (**III**).

To ascertain whether the solid-state structures were representative of their local minimum-energy conformations, gas-phase computational optimizations were performed on the new [13]-macrodilactones **I**–**III** and also compounds *a* and *e* in Fig. 3 ▸. The *pR* conformers from the X-ray data were optimized *via* DFT using the Schrödinger Maestro application *Jaguar* (Bochevarov *et al.*, 2013 ▸). Four different levels of theory, chosen because of their large number of basis functions and their inclusion of bromine orbitals, were used with the calculation, B3LYD-D3/CC-PVDC, M06-2X-D3/CC-PVDZ, B3LYD-D3/LACVP**, and M06-2X-D3/LACVP**. B3LYD-D3 was chosen because of its widespread use and M06-2X-D3 was chosen for its ability to accurately describe non-covalent inter­actions within the macrocycles (Grimme, 2011 ▸). Single point energy calculations were run on both the conformer from the crystallographic data and the conformer optimized at the B3LYD-D3/CC-PVDC level of theory. RMSD values comparing the 13 non-hydrogen atoms of the macrocycle ring were calculated comparing crystallographic structure and DFT-optimized conformers (Fig. 4 ▸). Values that express the average RMSD across the different DFT calculations ranged from 0.055 to 0.251 Å (Table S3 in the supporting information). The low values suggest that [13]-macrodilactones do not significantly change their conformations upon optimization.

## Supra­molecular features   

The crystal structures of **I**, **II** and **III** were searched for non-bonded inter­actions that may influence the measurements reported in Fig. 2 ▸. The program *Mercury* (Macrae, *et al.*, 2020 ▸) highlighted close contacts, which are defined as less than or equal to the sum of the van der Waals radii of atom pairs (Rowland *et al.*, 1996 ▸). In **I**, a contact of 3.188 (4) Å between the carbonyl oxygen O17 and methyl­ene carbon C11 in the mol­ecule generated by the *c*-glide operation was identified. Mol­ecules associated with this contact line up along [001]. In **II**, a Br⋯Br contact of 3.6466 (2) Å was identified where Br1 is near the crystallographic 2_1_ screw axis. Sequential applications of this symmetry operation generate a zigzag pattern of Br⋯Br contacts along [010]. In **III**, a contact of 3.386 (4) Å between C3 and the *para* carbon, C18, of the pendant phenyl ring is generated by the the 2_1_ screw axis and repeats along [001]. These distances fall well within what is observed in other solid state structures and no attractive inter­actions were found in **I**, **II**, or **III** (see Figures S1 to S7 in the supporting information).

## Synthesis and crystallization   

**Figure 5:**
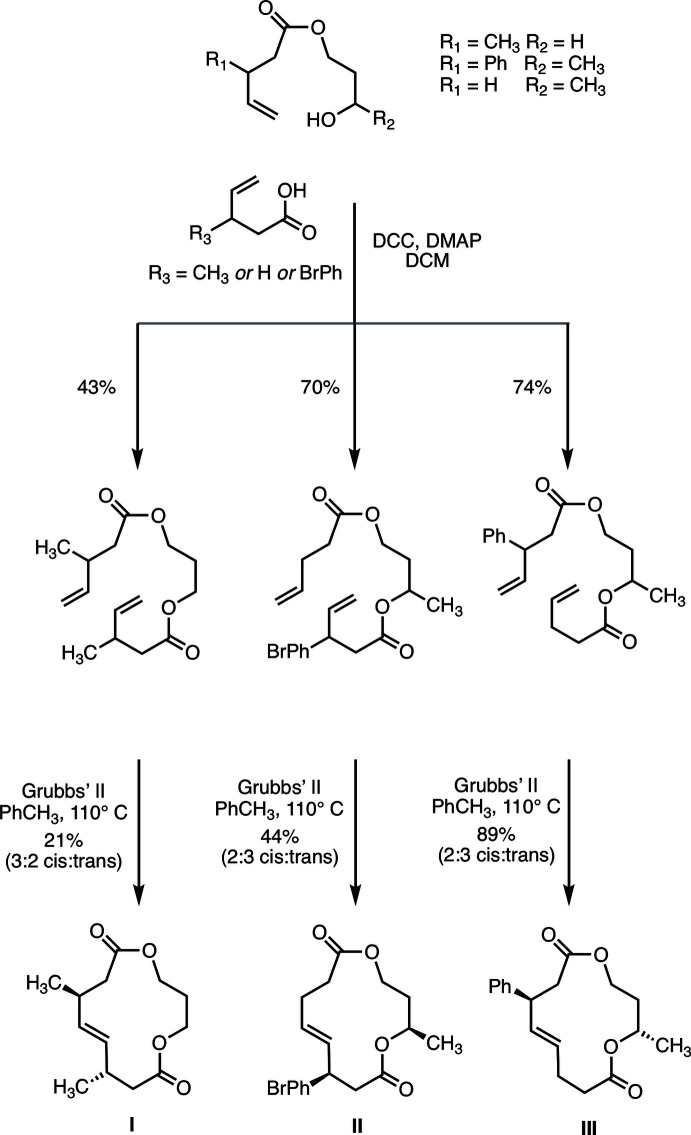
Synthesis of macrocycles **I**–**III**.

As shown in Fig. 5 ▸, [13]-macrodilactones **I**, **II**, and **III** were prepared by an established synthetic route that entailed sequential acyl­ation reactions, followed by macrocyclization *via* ring closing metathesis (RCM) (Magpusao *et al.*, 2015 ▸, 2016 ▸). Because the syntheses were not stereo-controlled and each of the new compounds contained two stereogenic centers, two diastereomeric products (each racemic) arose for each macrocycle. The diastereomers of **I**, **II**, and **III** in Fig. 4 ▸ are the ones that gave rise to the ‘ribbon’ conformers presented herein. See the supporting information for additional details on the synthesis of compounds **I**–**III**. Single crystals of the compounds were prepared by slow diffusion of hexane vapor into ethyl acetate:hexa­nes solutions of the compounds.

**General**

3-Bromo-phenyl-4-pentenoic acid, 3-phenyl-4-pentenoic acid and mono­acyl­ated 3-methyl-3-hy­droxy­propyl-4-pent­en­oate were prepared as previously described (Magpusao *et al.*, 2015 ▸). Unless stated otherwise, all acyl­ations were conducted at 273 K and allowed to warm to room temperature over 12 h. Reactions were monitored using TLC. Spots on TLC plates were visualized with UV light and *p*-anisaldehyde or ceric ammonium molybdate (CAM) stains. Chromatography was performed on silica gel and solvent systems were based on the *R*_f_ values. ^1^H NMR spectra were referenced to CDCl_3_ proton (δ H 7.27 ppm) and ^13^C NMR to the CDCl_3_ carbon (δ C 77.2 ppm).

*1,3-Di-(3-methyl-4-penteno­yloxy)-propane*

Into a 25 mL round-bottom flask were added di­cyclo­hexyl­carbodi­imide (DCC) (1.08 eq.), *N*,*N*-di­methyl­amino­pyridine (DMAP) (0.3 eq.) and di­chloro­methane (DCM) (7mL) and the solution was cooled to 273 K under nitro­gen. Then 3-methyl-4-pentenoic acid (1.0 eq.) was added and the mixture stirred at the same temperature for 30 minutes until a white suspension formed. A solution of 1,3-butane­diol (0.5 eq.) in DCM (3 mL) was then added and the mixture was stirred overnight at room temperature. After completion of the reaction, the mixture was filtered through a short pad of celite, rinsed with DCM, and solvent from the filtrates was removed under reduced pressure. The residue was then dissolved in cold ether to precipitate excess di­cyclo­hexyl urea (DCU), filtered through a pad of celite, and rinsed with additional ether. Ether from the combined filtrates was removed under reduced pressure and the residue was purified by column chromatography (10:90 EtOAc:Hex) to give a clear colorless oil (43%). *R*_f_ 0.47 (10:90 EtOAc:Hex); ^1^H NMR (CDCl_3_) 400 MHz δ 5.73 (*ddd*, *J* = 17.2, 10.2, 7.3 Hz, 2H), 4.96 (*dd*, *J* = 22.3, 17.2 Hz, 4H), 4.13 (*dd*, *J* = 6.33, 6.33 Hz, 4H), 2.65 (*s*, *J* = 7.0, 7.0, 7.0, 7.0, 7.0, 7.0, 7.0 Hz, 2H), 2.29 (*dddd* or *dq*, *J* = 22.1, 14.8, 14.8, 14.8 Hz, 4H), 1.94 (*dddd* or *dq*, *J* = 12.5, 6.2, 6.2, 6.2 Hz, 2H), 1.03 (*d*, *J* = 6.6 Hz, 6H); ^13^C NMR (CDCl_3_) 100 MHz δ 172.4, 142.5, 113.5, 61.0, 41.4, 34.6, 28.2, 19.9; TOF HRMS (DART) *m*/*z* calculated for C_15_H_24_O_4_ [*M*H]^+^ calculated 269.1753, found 269.1749.

**Sequential acyl­ation method**

To a 25 mL round-bottom flask was added DCM (7 mL), di­cyclo­hexyl­carbodi­imide (DCC) (1.08 eq.) and *N*,*N*-di­methyl­amino­pyridine (DMAP) (0.3 eq.) and the mixture was cooled to 273 K. 4-Pentenoic acid (1.0 eq.) was added and the mixture was stirred at the same temperature for 30 min until a white suspension was observed. Then, 1,3-propane­diol or 1,3-butane­diol (1.0 eq.) in DCM (3 mL) was added and the mixture was stirred overnight at room temperature. After completion of the reaction, the mixture was filtered through a short pad of celite, rinsed with DCM and solvent from the filtrates was removed under reduced pressure. The residue was then dissolved in cold ether to precipitate excess DCU, filtered through a pad of celite, and rinsed with additional ether. The crude residue was purified by silica gel column chromatography (15:85 EtOAc:Hex) to give the mono­acyl­ated product. The same procedure, where the mono-acyl­ated alcohol was used in place of the diol, was then followed for the second acyl­ation.

*1-[3-(p-Bromo­phen­yl)-4-penteno­yloxy]-1-methyl­propyl-4-pentenoate*

Synthesis followed the sequential acyl­ation method above to give the compound in 70% yield as a colorless oil. *R*_f_ 0.56 (hexa­nes: EtOAc 80:20); ^1^H NMR (CDCl_3_) 400 MHz δ 7.42 (*d*, *J* = 8.31 Hz, 2H), 7.09 (*d*, *J* = 8.3 Hz, 2H), 5.93 (*dddd*, *J* = 16.8, 10.3, 6.5, 1.7 Hz, 1H), 5.8 (*m*, 1H), 5.01 (*m*, 5H), 4.02 (*m*, 2H), 3.81 (*ddd*, *J* = 7.5, 7.5, 7.5 Hz, 1H), 2.73 (*m*, 1H), 2.64 (*ddd*, *J* = 15.3, 7.9, 0 Hz, 1H) 2.38 (*m*, 4H), 1.81 (*m*, 2H), 1.17 (*d*, *J* = 6.3 Hz, 2H), 1.12 (*d*, *J* = 6.3 Hz, 1H); ^13^C NMR (CDCl_3_) 100 MHz δ 173.1, 171.2, 141.5, 139.9, 136.8, 131.8, 129.6, 120.8, 115.7, 115.4, 68.3, 60.7, 45.3, 40.4, 34.9, 33.7, 29.0, 20.1.

*3-(3-Phenyl-4-penteno­yloxy)-1-methyl­propyl-4-pentenoate*

The synthesis followed the sequential acyl­ation method above to give the compound in 74% yield as a yellow oil. *R*_f_ 0.22 (hexa­nes: EtOAc 95:5); ^1^H NMR (CDCl_3_) 400 MHz δ 7.32 (*m*, 2H), 7.21 (*m*, 3H), 5.98 (*ddd*, *J* = 18.2, 10.14, 7.8 Hz, 1H), 5.81 (*m*, 1H), 5.0 (*m*, 5H), 4.21 (*m*, 2H), 3.86 [*ddd* (or *dt*), *J* = 7.4, 7.4, 7.4 Hz, 1H], 2.80 (*dd*, *J* = 15.1, 8.12 Hz, 1H), 2.70 (*dd*, *J* = 15.1, 7.4 Hz, 1H), 2.40 (*m*, 4H), 1.78 (*m*, 2H) 1.21 (*d*, 3H, *J* = 5.6 Hz); ^13^C NMR (CDCl_3_) 100 MHz δ 172.6, 171.8, 142.5, 140.4, 136.8, 128.7, 127.7, 126.8, 115.7, 115.0, 67.9, 60.9, 45.7, 40.3, 34.9, 33.9, 29.0, 20.2.

**General RCM Method**

Under an atmosphere of nitro­gen, Grubbs’ second-generation catalyst (0.10 eq.) was added to a solution of the diene in sufficient toluene so that the [diene] ≤ 10 m*M*. The mixture was heated to 383 K for 18 h. When the reaction was complete, the toluene was removed under reduced pressure to give a residue that was purified by column chromatography.

*trans-4,8-Dimethyl-1,10-dioxa­cyclo­tridec-5-ene-2,9-dione (**I**)*

Followed the general method of RCM in 21% overall yield (3:2 *cis*:*trans*) as a white solid. Compound **I** is the *trans* isomer; *R*_f_ 0.45 (hexa­nes: EtOAc 80:20) (higher *R*_f_, *trans*); ^1^HNMR (CDCl_3_) 400 MHz δ 5.80 (*dd*, *J* = 5.9, 2.6 Hz, 2H), 4.43 (*ddd*, *J* = 11.1, 8.8, 6.8 Hz, 2H), 3.91 (*dd*, *J* = 4.0, 4.0 Hz, 1H), 3.88 (*dd*, *J* = 4.0, 4.0 Hz, 1H), 2.61 (*m*, 2H), 2.30 (*dd*, *J* = 13.4, 3.2 Hz, 2H), 2.11 (*dd*, *J* = 12.7, 12.7 Hz, 2H), 2.00 (*dddd*, *J* = 9.5, 9.5, 4.0, 4.0 Hz, 2H), 1.03 (*d*, *J* = 6.9 Hz, 6H); ^13^C NMR (CDCl_3_) 100 MHz δ 173.2, 134.3, 59.8, 42.5, 35.9, 25.6, 21.6; TOF HRMS (DART) *m*/*z* [*M*+H]^+^ calculated for C_13_H_21_O_4_, calculated 241.1440, found 241.1440.

*cis-4-Phenyl-13-methyl-1,10-dioxa­cyclo­tridec-5-ene-2,9-di­one (**II**)*

Followed the general method of RCM in overall 44% yield (2:3 *cis*:*trans*). Compound **II** is the *cis* isomer and was isolated as a white solid. m.p. 370–374 K; *R*_f_ 0.46 (hexa­nes: EtOAc 80:20) (higher *R*_f_, *cis*); ^1^H NMR (CDCl_3_) 400 MHz δ 7.43 (*d*, *J* = 8.5 Hz, 2H), 7.10 (*d*, *J* = 8.5 Hz, 2H), 5.59 (*ddd*, *J* = 15.0, 8.7, 5.4 Hz, 1H), 5.44 (*dd*, *J* = 15.2, 9.0 Hz, 1H), 5.11 (*m*, 1H), 4.35 (*ddd*, *J* = 11.9, 11.9, 3.7 Hz, 1H), 3.95 (*ddd*, *J* = 11.3, 5.2, 2.4 Hz, 1H), 3.81 (*ddd*, *J* = 8.3, 8.3, 8.3 Hz, 1H), 2.55 (*m*, 2H), 2.32 (*m*, 4H), 2.04 (*m*, 1H), 1.81 (*dddd* or *ddt*, *J* = 14.6, 6.0, 3.3, 3.3 Hz, 1H), 1.30 (*d*, *J* = 6.2 Hz, 3H); ^13^C NMR (CDCl_3_) 100 MHz δ 174.0, 171.7, 142.2, 133.2, 132.0, 130.3, 129.0, 67.5, 60.5, 45.5, 41.5, 34.5, 33.5, 29.0, 20.7.

*trans-4-Phenyl-11-methyl-1,10-dioxa­cyclo­tridec-5-ene-2,9-di­one (**III**)*

Followed the general method of RCM in 89% overall yield 2:3 *cis*:*trans*). Compound **III** is the *trans* isomer, a yellow solid. m.p 359–362 K; *R*_f_ 0.45 (hexa­nes: EtOAc 80:20) (higher *R*_f_, *trans*); ^1^H NMR (CDCl_3_) 400 MHz δ 7.33 (*m*, 2H), 7.24 (*m*, 3H), 5.59 (*dd*, *J* = 15.5, 8.5 Hz, 1H), 5.53 (*ddd*, *J* = 15.9, 8.7, 5.1 Hz, 1H), 5.14 (*m*, 1H), 4.35 (*ddd*, *J* = 14.9, 11.4, 3.5 Hz, 1H), 3.94 (*ddd*, *J* = 12.4, 7.3, 1.3 Hz, 1H), 3.74 (*ddd*, *J* = 11.9, 8.8, 3.8, 1H), 2.61 (*dd*, *J* = 12.9, 12.9 Hz, 1H), 2.56 (*dd*, *J* = 12.6, 4.0 Hz, 1H), 2.42 (*m*, 2H), 2.29 (*m*, 1H), 2.20 (*m*, 1H), 2.1 (*dddd*, *J* = 18.6, 9.5, 5.7, 4.1 Hz, 1H), 1.8 (*m*, 1H), 1.31 (*d*, *J* = 6.19 Hz, 3H); ^13^C NMR (CDCl_3_) 100 MHz δ 173.0, 172.9, 143.3, 134.5, 129.0, 128.9, 127.1, 126.9, 66.8, 60.6, 47.1, 41.9, 34.3, 33.4, 28.1, 20.7.

## Refinement   

Crystal data, data collection and structure refinement details are summarized in Table 2 ▸. For all three structures, no evidence of disorder was found and no special restraints or constraints were required to achieve a stable refinement model. The hydrogen atoms were first found in the difference map, then generated geometrically and refined as riding atoms with C—H distances = 0.95–0.99 Å and *U*_iso_(H) = 1.2*U*_eq_(C) for CH and CH_2_ groups and *U*_iso_(H) = 1.5*U*_eq_(C) for CH_3_ groups.

## Supplementary Material

Crystal structure: contains datablock(s) global, I, II, III. DOI: 10.1107/S2056989020012037/mw2168sup1.cif

Structure factors: contains datablock(s) I. DOI: 10.1107/S2056989020012037/mw2168Isup2.hkl

Structure factors: contains datablock(s) II. DOI: 10.1107/S2056989020012037/mw2168IIsup3.hkl

Structure factors: contains datablock(s) III. DOI: 10.1107/S2056989020012037/mw2168IIIsup4.hkl

PDF document (Supplementary data associate with the manuscript: tables, computed structure coordinates, NMR spectra). DOI: 10.1107/S2056989020012037/mw2168sup5.pdf

CCDC references: 1944828, 1944827, 1944826

Additional supporting information:  crystallographic information; 3D view; checkCIF report

## Figures and Tables

**Table 1:** Substitution patterns and refcodes for [13]-macrodilactones Cpd. = compound identifier in Fig. 3 ▸, Conf. = conformer adopted in the crystal structure and Subs. = substituted positions on [13]-macrodilactone.

Entry	Cpd.	Conf.	Subs.	*cis/*trans**	Refcode	Citation
1	*a*	ribbon	11,13	*trans*	URILEO	Ma & Peczuh (2013 ▸)
2	*b*	ribbon	11 (mono)	–	KOHLAV	Fyvie & Peczuh (2008*a* ▸)
3	*c*	ribbon	3 (mono)	–	XUFKOA	Magpusao, Rutledge*et al.* (2015 ▸)
4	–	ribbon	D-*gluco*	*trans*	XOCWIW	Fyvie & Peczuh (2008*b* ▸)
5	*d*	ribbon	3,8	*trans*	XUFLAN	Magpusao, Rutledge *et al.* (2016 ▸)
6	*e*	ribbon	–	–	IJEHAI	Magpusao, Rutledge *et al.* (2016 ▸)
7	*f*	ribbon	3,11	*cis*	IJEHOW	Magpusao, Rutledge *et al.* (2016 ▸)
**8**	*g*	ribbon	4,13	*cis*	**II**	This work (CCDC 1944827)
**9**	*h*	ribbon	4,8	*trans*	**I**	This work (CCDC 1944826)
10	*i*	ribbon	4 (mono)	–	ECOYED	Rutledge, Hamlin *et al.* (2017 ▸)
11	*j*	ribbon	3,13	*trans*	IJEHEM	Magpusao, Rutledge *et al.* (2016 ▸)
10	–	*other*	4 (mono)	–	ECOYED	
12	–	*other*	11,13	*cis*	URILAK	Ma & Peczuh (2013 ▸)
3	*c*	*other*	3 (mono)	–	XUFKOA	
13	–	*other*	11,13	*cis*	URILAK	Ma & Peczuh (2013 ▸)
14	–	*other*	3,11	*trans*	IJEHUC	Magpusao, Rutledge *et al.* (2016 ▸)
**15**	k	ribbon	4,11	*trans*	**III**	This work (CCDC 1944828)
16	–	*other*	3,8	*cis*	XUFKUG	Magpusao, Rutledge *et al.* (2016 ▸)
17	–	*other*	8,11	*cis*	IJEHIQ	Magpusao, Rutledge *et al.* (2016 ▸)

**Table 2:** Experimental details

	(I)	(II)	(III)
Crystal data			
Chemical formula	C_13_H_20_O_4_	C_18_H_21_BrO_4_	C_18_H_22_O_4_
*M*_r_	240.29	381.26	302.35
Crystal system, space group	Monoclinic, *P*2_1_/*c*	Monoclinic, *P*2_1_/*n*	Orthorhombic, *P* *n* *a*2_1_
Temperature (K)	93	93	93
*a*, *b*, *c* (Å)	8.0547 (6), 18.7875 (14), 8.9660 (7)	15.3128 (3), 5.55594 (11), 20.5689 (4)	11.2952 (8), 20.9595 (15), 6.6840 (5)
α, β, γ (°)	90, 102.530 (6), 90	90, 95.7658 (18), 90	90, 90, 90
*V* (Å^3^)	1324.49 (18)	1741.08 (6)	1582.4 (2)
*Z*	4	4	4
Radiation type	Mo *K*α	Cu *K*α	Mo *K*α
μ (mm^−1^)	0.09	3.37	0.09
Crystal size (mm)	0.18 × 0.17 × 0.16	0.20 × 0.19 × 0.10	0.32 × 0.20 × 0.20

Data collection			
Diffractometer	Rigaku Mercury275R CCD	Rigaku Saturn 944+ CCD	Rigaku Mercury275R CCD
Absorption correction	Multi-scan (*REQAB*; Jacobson, 1998 ▸)	Multi-scan (*CrysAlis PRO*; Rigaku OD, 2015 ▸)	Multi-scan (*REQAB*; Jacobson, 1998 ▸)
*T*_min_, *T*_max_	0.705, 1.000	0.823, 1.000	0.815, 1.000
No. of measured, independent and observed [*I* > 2σ(*I*)] reflections	17742, 2341, 1835	59208, 3075, 3000	26669, 3664, 3410
*R*_int_	0.131	0.040	0.045
(sin θ/λ)_max_ (Å^−1^)	0.595	0.596	0.653

Refinement			
*R*[*F* ^2^ > 2σ(*F* ^2^)], *wR*(*F* ^2^), *S*	0.085, 0.230, 1.09	0.027, 0.062, 1.10	0.050, 0.128, 1.15
No. of reflections	2341	3075	3664
No. of parameters	156	209	201
No. of restraints	0	0	1
H-atom treatment	H-atom parameters constrained	H-atom parameters constrained	H-atom parameters constrained
Δρ_max_, Δρ_min_ (e Å^−3^)	0.53, −0.27	0.51, −0.52	0.26, −0.20
Absolute structure	–	–	Flack x determined using 1473 quotients [(*I* ^+^)−(*I* ^−^)]/[(*I* ^+^)+(*I* ^−^)] (Parsons *et al.*, 2013 ▸)
Absolute structure parameter	–	–	0.4 (5)

Computer programs: *CrystalClear-SM Expert* (Rigaku, 2011 ▸), *CrysAlis PRO* (Rigaku OD, 2015 ▸), *SHELXT2014/5* (Sheldrick, 2015*a* ▸), *SHELXL2014/7* and *SHELXL2013/2* (Sheldrick, 2015*b* ▸), *OLEX2* (Dolomanov *et al.*, 2009 ▸) and *CIFTAB2014/2* (Sheldrick, 2014 ▸).

## supplementary crystallographic information

### trans-4,8-Dimethyl-1,10-dioxacyclotridec-5-ene-2,9-dione (I) Crystal data

**Table:**

C_13_H_20_O_4_	*F*(000) = 520
*M**_r_* = 240.29	*D*_x_ = 1.205 Mg m^−^^3^
Monoclinic, *P*2_1_/*c*	Mo *K*α radiation, λ = 0.71073 Å
*a* = 8.0547 (6) Å	Cell parameters from 687 reflections
*b* = 18.7875 (14) Å	θ = 2.2–27.9°
*c* = 8.9660 (7) Å	µ = 0.09 mm^−^^1^
β = 102.530 (6)°	*T* = 93 K
*V* = 1324.49 (18) Å^3^	Prism, colorless
*Z* = 4	0.18 × 0.17 × 0.16 mm

### trans-4,8-Dimethyl-1,10-dioxacyclotridec-5-ene-2,9-dione (I) Data collection

**Table:**

Rigaku Mercury275R CCD diffractometer	2341 independent reflections
Radiation source: Sealed Tube	1835 reflections with *I* > 2σ(*I*)
Detector resolution: 6.8 pixels mm^-1^	*R*_int_ = 0.131
ω scans	θ_max_ = 25.0°, θ_min_ = 2.2°
Absorption correction: multi-scan (*REQAB*; Jacobson, 1998)	*h* = −9→9
*T*_min_ = 0.705, *T*_max_ = 1.000	*k* = −22→22
17742 measured reflections	*l* = −10→10

### trans-4,8-Dimethyl-1,10-dioxacyclotridec-5-ene-2,9-dione (I) Refinement

**Table:**

Refinement on *F*^2^	Primary atom site location: dual
Least-squares matrix: full	Secondary atom site location: difference Fourier map
*R*[*F*^2^ > 2σ(*F*^2^)] = 0.085	Hydrogen site location: inferred from neighbouring sites
*wR*(*F*^2^) = 0.230	H-atom parameters constrained
*S* = 1.09	*w* = 1/[σ^2^(*F*_o_^2^) + (0.1386*P*)^2^ + 0.4739*P*] where *P* = (*F*_o_^2^ + 2*F*_c_^2^)/3
2341 reflections	(Δ/σ)_max_ < 0.001
156 parameters	Δρ_max_ = 0.53 e Å^−^^3^
0 restraints	Δρ_min_ = −0.27 e Å^−^^3^

### trans-4,8-Dimethyl-1,10-dioxacyclotridec-5-ene-2,9-dione (I) Special details

**Table:**

Geometry. All esds (except the esd in the dihedral angle between two l.s. planes) are estimated using the full covariance matrix. The cell esds are taken into account individually in the estimation of esds in distances, angles and torsion angles; correlations between esds in cell parameters are only used when they are defined by crystal symmetry. An approximate (isotropic) treatment of cell esds is used for estimating esds involving l.s. planes.
Refinement. Refinement of F^2^ against ALL reflections. The weighted R-factor wR and goodness of fit S are based on F^2^, conventional R-factors R are based on F, with F set to zero for negative F^2^. The threshold expression of F^2^ > 2sigma(F^2^) is used only for calculating R-factors(gt) etc. and is not relevant to the choice of reflections for refinement. R-factors based on F^2^ are statistically about twice as large as those based on F, and R- factors based on ALL data will be even larger. The hydrogen atoms were first found in the difference map, then generated geometrically and refined as riding atoms with C-H distances = 0.95 - 0.99 angstroms and Uiso(H) = 1.2 times Ueq(C) for CH and CH2 groups and Uiso(H) = 1.5 times Ueq(C) for CH3 groups.

### trans-4,8-Dimethyl-1,10-dioxacyclotridec-5-ene-2,9-dione (I) Fractional atomic coordinates and isotropic or equivalent isotropic displacement parameters (Å^2^)

**Table:**

	*x*	*y*	*z*	*U*_iso_*/*U*_eq_	
O1	0.5209 (3)	0.33574 (11)	0.6049 (2)	0.0443 (6)	
O10	0.8844 (3)	0.38480 (10)	0.5944 (2)	0.0426 (6)	
O14	0.3382 (3)	0.42433 (13)	0.5285 (3)	0.0583 (7)	
O17	0.9157 (3)	0.31352 (11)	0.4047 (2)	0.0441 (6)	
C2	0.3918 (4)	0.36655 (17)	0.5079 (3)	0.0419 (7)	
C3	0.3224 (4)	0.32154 (16)	0.3709 (3)	0.0409 (7)	
H3A	0.2054	0.3064	0.3736	0.049*	
H3B	0.3932	0.2782	0.3740	0.049*	
C4	0.3199 (4)	0.36195 (15)	0.2221 (3)	0.0387 (7)	
H4	0.2743	0.4108	0.2317	0.046*	
C5	0.4992 (3)	0.36868 (15)	0.1969 (3)	0.0378 (7)	
H5	0.5469	0.3282	0.1584	0.045*	
C6	0.5933 (4)	0.42558 (15)	0.2237 (3)	0.0372 (7)	
H6	0.5432	0.4665	0.2580	0.045*	
C7	0.7736 (4)	0.43256 (15)	0.2055 (3)	0.0384 (7)	
H7	0.8039	0.3891	0.1526	0.046*	
C8	0.8940 (4)	0.43846 (15)	0.3605 (3)	0.0401 (7)	
H8A	1.0097	0.4494	0.3460	0.048*	
H8B	0.8573	0.4784	0.4179	0.048*	
C9	0.8998 (3)	0.37194 (15)	0.4516 (3)	0.0369 (7)	
C11	0.8732 (4)	0.32312 (17)	0.6890 (3)	0.0450 (8)	
H11A	0.9884	0.3077	0.7422	0.054*	
H11B	0.8174	0.2832	0.6252	0.054*	
C12	0.7703 (4)	0.34428 (18)	0.8036 (3)	0.0464 (8)	
H12A	0.7449	0.3013	0.8584	0.056*	
H12B	0.8387	0.3768	0.8797	0.056*	
C13	0.6064 (4)	0.38033 (17)	0.7309 (3)	0.0460 (8)	
H13A	0.5341	0.3861	0.8064	0.055*	
H13B	0.6293	0.4280	0.6928	0.055*	
C15	0.2026 (4)	0.32451 (17)	0.0917 (4)	0.0468 (8)	
H15A	0.2431	0.2758	0.0830	0.070*	
H15B	0.2010	0.3503	−0.0037	0.070*	
H15C	0.0874	0.3231	0.1109	0.070*	
C16	0.7922 (4)	0.49735 (17)	0.1094 (4)	0.0498 (8)	
H16A	0.7628	0.5403	0.1602	0.075*	
H16B	0.7159	0.4928	0.0087	0.075*	
H16C	0.9100	0.5008	0.0974	0.075*	

### trans-4,8-Dimethyl-1,10-dioxacyclotridec-5-ene-2,9-dione (I) Atomic displacement parameters (Å^2^)

**Table:**

	*U*^11^	*U*^22^	*U*^33^	*U*^12^	*U*^13^	*U*^23^
O1	0.0427 (12)	0.0569 (13)	0.0360 (11)	0.0046 (9)	0.0147 (10)	−0.0001 (9)
O10	0.0475 (12)	0.0532 (12)	0.0304 (11)	0.0040 (9)	0.0158 (9)	−0.0037 (8)
O14	0.0538 (14)	0.0732 (16)	0.0481 (14)	0.0204 (12)	0.0117 (11)	−0.0121 (11)
O17	0.0451 (12)	0.0523 (13)	0.0410 (12)	0.0014 (9)	0.0231 (10)	−0.0055 (9)
C2	0.0361 (15)	0.0590 (19)	0.0358 (16)	0.0042 (13)	0.0194 (13)	0.0013 (13)
C3	0.0316 (15)	0.0555 (18)	0.0400 (17)	0.0003 (12)	0.0176 (13)	0.0009 (12)
C4	0.0322 (15)	0.0514 (17)	0.0364 (15)	0.0003 (12)	0.0161 (12)	−0.0003 (12)
C5	0.0335 (15)	0.0539 (17)	0.0308 (14)	0.0020 (12)	0.0175 (12)	−0.0032 (12)
C6	0.0385 (15)	0.0481 (16)	0.0297 (14)	0.0040 (12)	0.0179 (12)	0.0002 (11)
C7	0.0354 (15)	0.0519 (17)	0.0337 (15)	−0.0015 (12)	0.0202 (12)	−0.0026 (11)
C8	0.0352 (15)	0.0516 (17)	0.0391 (16)	−0.0020 (12)	0.0205 (13)	−0.0053 (12)
C9	0.0248 (13)	0.0525 (18)	0.0360 (15)	0.0018 (11)	0.0120 (12)	−0.0047 (12)
C11	0.0493 (18)	0.0552 (18)	0.0341 (16)	0.0086 (13)	0.0170 (14)	0.0017 (12)
C12	0.0477 (18)	0.063 (2)	0.0306 (15)	0.0076 (14)	0.0136 (14)	−0.0013 (13)
C13	0.0502 (18)	0.0589 (19)	0.0320 (15)	0.0054 (14)	0.0161 (14)	−0.0041 (12)
C15	0.0391 (17)	0.0627 (19)	0.0418 (17)	−0.0038 (14)	0.0161 (14)	−0.0009 (14)
C16	0.0486 (19)	0.065 (2)	0.0422 (17)	−0.0030 (15)	0.0247 (16)	0.0033 (14)

### trans-4,8-Dimethyl-1,10-dioxacyclotridec-5-ene-2,9-dione (I) Geometric parameters (Å, º)

**Table:**

O1—C2	1.334 (4)	C7—H7	1.0000
O1—C13	1.454 (4)	C8—C9	1.488 (4)
O10—C9	1.335 (3)	C8—H8A	0.9900
O10—C11	1.450 (4)	C8—H8B	0.9900
O14—C2	1.197 (4)	C11—C12	1.506 (4)
O17—C9	1.192 (3)	C11—H11A	0.9900
C2—C3	1.496 (4)	C11—H11B	0.9900
C3—C4	1.532 (4)	C12—C13	1.501 (4)
C3—H3A	0.9900	C12—H12A	0.9900
C3—H3B	0.9900	C12—H12B	0.9900
C4—C15	1.508 (4)	C13—H13A	0.9900
C4—C5	1.515 (4)	C13—H13B	0.9900
C4—H4	1.0000	C15—H15A	0.9800
C5—C6	1.302 (4)	C15—H15B	0.9800
C5—H5	0.9500	C15—H15C	0.9800
C6—C7	1.502 (4)	C16—H16A	0.9800
C6—H6	0.9500	C16—H16B	0.9800
C7—C8	1.516 (4)	C16—H16C	0.9800
C7—C16	1.517 (4)		
			
C2—O1—C13	115.3 (2)	H8A—C8—H8B	107.9
C9—O10—C11	116.5 (2)	O17—C9—O10	123.0 (3)
O14—C2—O1	123.3 (3)	O17—C9—C8	124.9 (3)
O14—C2—C3	123.8 (3)	O10—C9—C8	112.1 (2)
O1—C2—C3	112.9 (3)	O10—C11—C12	107.5 (2)
C2—C3—C4	111.5 (2)	O10—C11—H11A	110.2
C2—C3—H3A	109.3	C12—C11—H11A	110.2
C4—C3—H3A	109.3	O10—C11—H11B	110.2
C2—C3—H3B	109.3	C12—C11—H11B	110.2
C4—C3—H3B	109.3	H11A—C11—H11B	108.5
H3A—C3—H3B	108.0	C13—C12—C11	112.7 (2)
C15—C4—C5	112.4 (2)	C13—C12—H12A	109.1
C15—C4—C3	109.4 (2)	C11—C12—H12A	109.1
C5—C4—C3	109.8 (2)	C13—C12—H12B	109.1
C15—C4—H4	108.4	C11—C12—H12B	109.1
C5—C4—H4	108.4	H12A—C12—H12B	107.8
C3—C4—H4	108.4	O1—C13—C12	107.5 (2)
C6—C5—C4	125.2 (2)	O1—C13—H13A	110.2
C6—C5—H5	117.4	C12—C13—H13A	110.2
C4—C5—H5	117.4	O1—C13—H13B	110.2
C5—C6—C7	126.1 (3)	C12—C13—H13B	110.2
C5—C6—H6	117.0	H13A—C13—H13B	108.5
C7—C6—H6	117.0	C4—C15—H15A	109.5
C6—C7—C8	110.4 (2)	C4—C15—H15B	109.5
C6—C7—C16	110.4 (2)	H15A—C15—H15B	109.5
C8—C7—C16	109.8 (2)	C4—C15—H15C	109.5
C6—C7—H7	108.7	H15A—C15—H15C	109.5
C8—C7—H7	108.7	H15B—C15—H15C	109.5
C16—C7—H7	108.7	C7—C16—H16A	109.5
C9—C8—C7	112.3 (2)	C7—C16—H16B	109.5
C9—C8—H8A	109.1	H16A—C16—H16B	109.5
C7—C8—H8A	109.1	C7—C16—H16C	109.5
C9—C8—H8B	109.1	H16A—C16—H16C	109.5
C7—C8—H8B	109.1	H16B—C16—H16C	109.5
			
C13—O1—C2—O14	−6.6 (4)	C6—C7—C8—C9	66.3 (3)
C13—O1—C2—C3	173.1 (2)	C16—C7—C8—C9	−171.8 (2)
O14—C2—C3—C4	53.8 (4)	C11—O10—C9—O17	−5.1 (4)
O1—C2—C3—C4	−125.8 (2)	C11—O10—C9—C8	174.7 (2)
C2—C3—C4—C15	−162.7 (2)	C7—C8—C9—O17	47.6 (4)
C2—C3—C4—C5	73.6 (3)	C7—C8—C9—O10	−132.2 (2)
C15—C4—C5—C6	136.5 (3)	C9—O10—C11—C12	−151.1 (3)
C3—C4—C5—C6	−101.5 (3)	O10—C11—C12—C13	49.6 (4)
C4—C5—C6—C7	177.4 (3)	C2—O1—C13—C12	−166.3 (2)
C5—C6—C7—C8	−109.7 (3)	C11—C12—C13—O1	50.5 (4)
C5—C6—C7—C16	128.7 (3)		

### cis-4-(4-Bromophenyl)-13-methyl-1,10-dioxacyclotridec-5-ene-2,9-dione (II) Crystal data

**Table:**

C_18_H_21_BrO_4_	*F*(000) = 784
*M**_r_* = 381.26	*D*_x_ = 1.454 Mg m^−^^3^
Monoclinic, *P*2_1_/*n*	Cu *K*α radiation, λ = 1.54184 Å
*a* = 15.3128 (3) Å	Cell parameters from 41470 reflections
*b* = 5.55594 (11) Å	θ = 2.1–66.6°
*c* = 20.5689 (4) Å	µ = 3.37 mm^−^^1^
β = 95.7658 (18)°	*T* = 93 K
*V* = 1741.08 (6) Å^3^	Block, colorless
*Z* = 4	0.20 × 0.19 × 0.10 mm

### cis-4-(4-Bromophenyl)-13-methyl-1,10-dioxacyclotridec-5-ene-2,9-dione (II) Data collection

**Table:**

Rigaku Saturn 944+ CCD diffractometer	3075 independent reflections
Radiation source: microfocus rotating anode	3000 reflections with *I* > 2σ(*I*)
Detector resolution: 22.2 pixels mm^-1^	*R*_int_ = 0.040
ω scans	θ_max_ = 66.8°, θ_min_ = 3.4°
Absorption correction: multi-scan (CrysAlisPro; Rigaku OD, 2015)	*h* = −18→18
*T*_min_ = 0.823, *T*_max_ = 1.000	*k* = −6→6
59208 measured reflections	*l* = −24→24

### cis-4-(4-Bromophenyl)-13-methyl-1,10-dioxacyclotridec-5-ene-2,9-dione (II) Refinement

**Table:**

Refinement on *F*^2^	Primary atom site location: structure-invariant direct methods
Least-squares matrix: full	Secondary atom site location: difference Fourier map
*R*[*F*^2^ > 2σ(*F*^2^)] = 0.027	Hydrogen site location: inferred from neighbouring sites
*wR*(*F*^2^) = 0.062	H-atom parameters constrained
*S* = 1.10	*w* = 1/[σ^2^(*F*_o_^2^) + (0.0186*P*)^2^ + 2.082*P*] where *P* = (*F*_o_^2^ + 2*F*_c_^2^)/3
3075 reflections	(Δ/σ)_max_ = 0.001
209 parameters	Δρ_max_ = 0.51 e Å^−^^3^
0 restraints	Δρ_min_ = −0.52 e Å^−^^3^

### cis-4-(4-Bromophenyl)-13-methyl-1,10-dioxacyclotridec-5-ene-2,9-dione (II) Special details

**Table:**

Geometry. All esds (except the esd in the dihedral angle between two l.s. planes) are estimated using the full covariance matrix. The cell esds are taken into account individually in the estimation of esds in distances, angles and torsion angles; correlations between esds in cell parameters are only used when they are defined by crystal symmetry. An approximate (isotropic) treatment of cell esds is used for estimating esds involving l.s. planes.
Refinement. Refinement of F^2^ against ALL reflections. The weighted R-factor wR and goodness of fit S are based on F^2^, conventional R-factors R are based on F, with F set to zero for negative F^2^. The threshold expression of F^2^ > 2sigma(F^2^) is used only for calculating R-factors(gt) etc. and is not relevant to the choice of reflections for refinement. R-factors based on F^2^ are statistically about twice as large as those based on F, and R- factors based on ALL data will be even larger. The hydrogen atoms were first found in the difference map, then generated geometrically and refined as riding atoms with C-H distances = 0.95 - 0.99 angstroms and Uiso(H) = 1.2 times Ueq(C) for CH and CH2 groups and Uiso(H) = 1.5 times Ueq(C) for CH3 groups.

### cis-4-(4-Bromophenyl)-13-methyl-1,10-dioxacyclotridec-5-ene-2,9-dione (II) Fractional atomic coordinates and isotropic or equivalent isotropic displacement parameters (Å^2^)

**Table:**

	*x*	*y*	*z*	*U*_iso_*/*U*_eq_	
Br1	0.67994 (2)	1.25106 (4)	0.26935 (2)	0.02712 (8)	
O1	0.27479 (8)	0.5670 (2)	0.52299 (6)	0.0222 (3)	
O10	0.08625 (9)	0.6437 (3)	0.45484 (7)	0.0297 (3)	
O15	0.32471 (11)	0.2793 (3)	0.45930 (8)	0.0361 (4)	
O22	0.08714 (12)	1.0133 (3)	0.40964 (8)	0.0466 (4)	
C2	0.32629 (13)	0.4847 (4)	0.47904 (9)	0.0246 (4)	
C3	0.38595 (13)	0.6777 (4)	0.45684 (9)	0.0253 (4)	
H3A	0.4470	0.6480	0.4759	0.030*	
H3B	0.3674	0.8369	0.4721	0.030*	
C4	0.38196 (12)	0.6768 (4)	0.38145 (9)	0.0245 (4)	
H4	0.3863	0.5058	0.3669	0.029*	
C5	0.29466 (13)	0.7741 (4)	0.35257 (9)	0.0271 (4)	
H5	0.2847	0.9422	0.3559	0.032*	
C6	0.23075 (14)	0.6403 (4)	0.32272 (10)	0.0311 (5)	
H6	0.2416	0.4733	0.3177	0.037*	
C7	0.14240 (14)	0.7363 (4)	0.29646 (10)	0.0334 (5)	
H7A	0.1272	0.6740	0.2517	0.040*	
H7B	0.1452	0.9140	0.2940	0.040*	
C8	0.07030 (13)	0.6635 (5)	0.33969 (10)	0.0339 (5)	
H8A	0.0120	0.7011	0.3167	0.041*	
H8B	0.0731	0.4879	0.3478	0.041*	
C9	0.08167 (14)	0.7959 (4)	0.40389 (11)	0.0316 (5)	
C11	0.10483 (14)	0.7529 (4)	0.51898 (10)	0.0313 (5)	
H11A	0.0512	0.8289	0.5328	0.038*	
H11B	0.1506	0.8782	0.5178	0.038*	
C12	0.13630 (13)	0.5550 (4)	0.56615 (9)	0.0275 (4)	
H12A	0.0858	0.4510	0.5737	0.033*	
H12B	0.1582	0.6286	0.6085	0.033*	
C13	0.20832 (12)	0.3996 (4)	0.54275 (9)	0.0231 (4)	
H13	0.1840	0.3031	0.5041	0.028*	
C14	0.24961 (14)	0.2320 (4)	0.59525 (10)	0.0290 (5)	
H14A	0.2794	0.3270	0.6310	0.043*	
H14B	0.2923	0.1275	0.5767	0.043*	
H14C	0.2038	0.1331	0.6120	0.043*	
C16	0.45741 (12)	0.8154 (4)	0.35657 (9)	0.0213 (4)	
C17	0.48886 (12)	1.0298 (4)	0.38536 (9)	0.0239 (4)	
H17	0.4642	1.0888	0.4228	0.029*	
C18	0.55561 (13)	1.1589 (4)	0.36022 (9)	0.0240 (4)	
H18	0.5769	1.3044	0.3803	0.029*	
C19	0.59046 (12)	1.0712 (4)	0.30541 (9)	0.0217 (4)	
C20	0.56177 (13)	0.8585 (4)	0.27595 (9)	0.0241 (4)	
H20	0.5871	0.7998	0.2387	0.029*	
C21	0.49528 (13)	0.7323 (4)	0.30178 (9)	0.0239 (4)	
H21	0.4750	0.5859	0.2818	0.029*	

### cis-4-(4-Bromophenyl)-13-methyl-1,10-dioxacyclotridec-5-ene-2,9-dione (II) Atomic displacement parameters (Å^2^)

**Table:**

	*U*^11^	*U*^22^	*U*^33^	*U*^12^	*U*^13^	*U*^23^
Br1	0.02422 (12)	0.02824 (13)	0.03034 (13)	−0.00158 (8)	0.00974 (8)	0.00148 (8)
O1	0.0221 (6)	0.0222 (7)	0.0230 (6)	−0.0016 (6)	0.0054 (5)	−0.0003 (5)
O10	0.0267 (7)	0.0356 (8)	0.0258 (7)	−0.0019 (6)	−0.0033 (6)	−0.0008 (6)
O15	0.0445 (9)	0.0246 (8)	0.0427 (9)	0.0027 (7)	0.0216 (7)	0.0000 (7)
O22	0.0656 (12)	0.0349 (10)	0.0395 (9)	0.0091 (9)	0.0059 (8)	−0.0002 (8)
C2	0.0246 (10)	0.0255 (11)	0.0242 (9)	0.0040 (8)	0.0048 (8)	0.0025 (8)
C3	0.0221 (9)	0.0291 (11)	0.0250 (10)	−0.0001 (8)	0.0035 (8)	0.0038 (8)
C4	0.0230 (10)	0.0272 (11)	0.0237 (10)	−0.0003 (8)	0.0041 (8)	0.0008 (8)
C5	0.0228 (10)	0.0340 (12)	0.0245 (10)	0.0015 (9)	0.0030 (8)	0.0028 (9)
C6	0.0331 (11)	0.0340 (12)	0.0264 (10)	0.0019 (10)	0.0037 (8)	−0.0019 (9)
C7	0.0300 (11)	0.0421 (13)	0.0268 (10)	−0.0016 (10)	−0.0027 (9)	−0.0007 (10)
C8	0.0237 (10)	0.0476 (14)	0.0292 (11)	−0.0040 (10)	−0.0039 (8)	−0.0011 (10)
C9	0.0222 (10)	0.0410 (14)	0.0315 (11)	0.0060 (9)	0.0026 (8)	0.0016 (10)
C11	0.0299 (11)	0.0360 (12)	0.0277 (11)	0.0070 (10)	0.0017 (9)	−0.0070 (9)
C12	0.0235 (10)	0.0353 (12)	0.0241 (10)	−0.0010 (9)	0.0047 (8)	−0.0027 (9)
C13	0.0241 (9)	0.0239 (10)	0.0214 (9)	−0.0055 (8)	0.0034 (7)	0.0004 (8)
C14	0.0356 (11)	0.0287 (11)	0.0225 (10)	−0.0013 (9)	0.0023 (8)	0.0034 (9)
C16	0.0194 (9)	0.0227 (10)	0.0217 (9)	0.0025 (8)	0.0014 (7)	0.0035 (8)
C17	0.0234 (9)	0.0260 (10)	0.0230 (9)	0.0023 (8)	0.0065 (7)	−0.0018 (8)
C18	0.0246 (10)	0.0220 (10)	0.0254 (9)	0.0000 (8)	0.0025 (8)	−0.0018 (8)
C19	0.0181 (9)	0.0245 (10)	0.0226 (9)	0.0021 (8)	0.0024 (7)	0.0043 (8)
C20	0.0264 (10)	0.0257 (10)	0.0207 (9)	0.0044 (8)	0.0052 (8)	−0.0004 (8)
C21	0.0270 (10)	0.0221 (10)	0.0223 (9)	0.0002 (8)	0.0013 (8)	−0.0006 (8)

### cis-4-(4-Bromophenyl)-13-methyl-1,10-dioxacyclotridec-5-ene-2,9-dione (II) Geometric parameters (Å, º)

**Table:**

Br1—C19	1.9053 (19)	C8—H8B	0.9900
O1—C2	1.338 (2)	C11—C12	1.513 (3)
O1—C13	1.466 (2)	C11—H11A	0.9900
O10—C9	1.343 (3)	C11—H11B	0.9900
O10—C11	1.454 (2)	C12—C13	1.516 (3)
O15—C2	1.210 (3)	C12—H12A	0.9900
O22—C9	1.216 (3)	C12—H12B	0.9900
C2—C3	1.509 (3)	C13—C14	1.515 (3)
C3—C4	1.546 (3)	C13—H13	1.0000
C3—H3A	0.9900	C14—H14A	0.9800
C3—H3B	0.9900	C14—H14B	0.9800
C4—C5	1.508 (3)	C14—H14C	0.9800
C4—C16	1.519 (3)	C16—C17	1.395 (3)
C4—H4	1.0000	C16—C21	1.397 (3)
C5—C6	1.329 (3)	C17—C18	1.390 (3)
C5—H5	0.9500	C17—H17	0.9500
C6—C7	1.503 (3)	C18—C19	1.384 (3)
C6—H6	0.9500	C18—H18	0.9500
C7—C8	1.540 (3)	C19—C20	1.379 (3)
C7—H7A	0.9900	C20—C21	1.385 (3)
C7—H7B	0.9900	C20—H20	0.9500
C8—C9	1.506 (3)	C21—H21	0.9500
C8—H8A	0.9900		
			
C2—O1—C13	116.31 (15)	C12—C11—H11A	110.2
C9—O10—C11	115.81 (18)	O10—C11—H11B	110.2
O15—C2—O1	123.73 (19)	C12—C11—H11B	110.2
O15—C2—C3	124.10 (18)	H11A—C11—H11B	108.5
O1—C2—C3	112.16 (17)	C11—C12—C13	113.90 (16)
C2—C3—C4	109.70 (17)	C11—C12—H12A	108.8
C2—C3—H3A	109.7	C13—C12—H12A	108.8
C4—C3—H3A	109.7	C11—C12—H12B	108.8
C2—C3—H3B	109.7	C13—C12—H12B	108.8
C4—C3—H3B	109.7	H12A—C12—H12B	107.7
H3A—C3—H3B	108.2	O1—C13—C14	109.62 (15)
C5—C4—C16	111.08 (17)	O1—C13—C12	105.93 (16)
C5—C4—C3	109.77 (16)	C14—C13—C12	112.85 (16)
C16—C4—C3	112.43 (16)	O1—C13—H13	109.5
C5—C4—H4	107.8	C14—C13—H13	109.5
C16—C4—H4	107.8	C12—C13—H13	109.5
C3—C4—H4	107.8	C13—C14—H14A	109.5
C6—C5—C4	124.3 (2)	C13—C14—H14B	109.5
C6—C5—H5	117.8	H14A—C14—H14B	109.5
C4—C5—H5	117.8	C13—C14—H14C	109.5
C5—C6—C7	124.2 (2)	H14A—C14—H14C	109.5
C5—C6—H6	117.9	H14B—C14—H14C	109.5
C7—C6—H6	117.9	C17—C16—C21	118.13 (18)
C6—C7—C8	111.78 (18)	C17—C16—C4	122.13 (17)
C6—C7—H7A	109.3	C21—C16—C4	119.71 (18)
C8—C7—H7A	109.3	C18—C17—C16	121.21 (18)
C6—C7—H7B	109.3	C18—C17—H17	119.4
C8—C7—H7B	109.3	C16—C17—H17	119.4
H7A—C7—H7B	107.9	C19—C18—C17	118.67 (19)
C9—C8—C7	110.59 (18)	C19—C18—H18	120.7
C9—C8—H8A	109.5	C17—C18—H18	120.7
C7—C8—H8A	109.5	C20—C19—C18	121.85 (18)
C9—C8—H8B	109.5	C20—C19—Br1	119.21 (14)
C7—C8—H8B	109.5	C18—C19—Br1	118.94 (15)
H8A—C8—H8B	108.1	C19—C20—C21	118.62 (18)
O22—C9—O10	123.5 (2)	C19—C20—H20	120.7
O22—C9—C8	124.8 (2)	C21—C20—H20	120.7
O10—C9—C8	111.6 (2)	C20—C21—C16	121.52 (19)
O10—C11—C12	107.42 (17)	C20—C21—H21	119.2
O10—C11—H11A	110.2	C16—C21—H21	119.2
			
C13—O1—C2—O15	−5.6 (3)	C2—O1—C13—C12	−155.41 (15)
C13—O1—C2—C3	174.13 (15)	C11—C12—C13—O1	50.0 (2)
O15—C2—C3—C4	47.6 (3)	C11—C12—C13—C14	169.98 (18)
O1—C2—C3—C4	−132.13 (17)	C5—C4—C16—C17	84.5 (2)
C2—C3—C4—C5	71.6 (2)	C3—C4—C16—C17	−38.9 (3)
C2—C3—C4—C16	−164.23 (16)	C5—C4—C16—C21	−93.3 (2)
C16—C4—C5—C6	128.0 (2)	C3—C4—C16—C21	143.29 (18)
C3—C4—C5—C6	−107.0 (2)	C21—C16—C17—C18	0.4 (3)
C4—C5—C6—C7	177.34 (19)	C4—C16—C17—C18	−177.39 (18)
C5—C6—C7—C8	−105.8 (3)	C16—C17—C18—C19	0.3 (3)
C6—C7—C8—C9	70.3 (3)	C17—C18—C19—C20	−1.1 (3)
C11—O10—C9—O22	−4.3 (3)	C17—C18—C19—Br1	178.45 (14)
C11—O10—C9—C8	174.89 (16)	C18—C19—C20—C21	1.0 (3)
C7—C8—C9—O22	54.0 (3)	Br1—C19—C20—C21	−178.54 (14)
C7—C8—C9—O10	−125.2 (2)	C19—C20—C21—C16	−0.2 (3)
C9—O10—C11—C12	−161.83 (17)	C17—C16—C21—C20	−0.5 (3)
O10—C11—C12—C13	49.7 (2)	C4—C16—C21—C20	177.35 (18)
C2—O1—C13—C14	82.6 (2)		

### trans-11-Methyl-4-phenyl-1,10-dioxacyclotridec-5-ene-2,9-dione (III) Crystal data

**Table:**

C_18_H_22_O_4_	*D*_x_ = 1.269 Mg m^−^^3^
*M**_r_* = 302.35	Mo *K*α radiation, λ = 0.71073 Å
Orthorhombic, *P**n**a*2_1_	Cell parameters from 576 reflections
*a* = 11.2952 (8) Å	θ = 2.6–27.5°
*b* = 20.9595 (15) Å	µ = 0.09 mm^−^^1^
*c* = 6.6840 (5) Å	*T* = 93 K
*V* = 1582.4 (2) Å^3^	Prism, colorless
*Z* = 4	0.32 × 0.20 × 0.20 mm
*F*(000) = 648	

### trans-11-Methyl-4-phenyl-1,10-dioxacyclotridec-5-ene-2,9-dione (III) Data collection

**Table:**

Rigaku Mercury275R CCD diffractometer	3664 independent reflections
Radiation source: Sealed Tube	3410 reflections with *I* > 2σ(*I*)
Detector resolution: 6.8 pixels mm^-1^	*R*_int_ = 0.045
ω scans	θ_max_ = 27.7°, θ_min_ = 2.7°
Absorption correction: multi-scan (*REQAB*; Jacobson, 1998)	*h* = −14→14
*T*_min_ = 0.815, *T*_max_ = 1.000	*k* = −27→27
26669 measured reflections	*l* = −8→8

### trans-11-Methyl-4-phenyl-1,10-dioxacyclotridec-5-ene-2,9-dione (III) Refinement

**Table:**

Refinement on *F*^2^	Hydrogen site location: inferred from neighbouring sites
Least-squares matrix: full	H-atom parameters constrained
*R*[*F*^2^ > 2σ(*F*^2^)] = 0.050	*w* = 1/[σ^2^(*F*_o_^2^) + (0.0578*P*)^2^ + 0.8966*P*] where *P* = (*F*_o_^2^ + 2*F*_c_^2^)/3
*wR*(*F*^2^) = 0.128	(Δ/σ)_max_ < 0.001
*S* = 1.15	Δρ_max_ = 0.26 e Å^−^^3^
3664 reflections	Δρ_min_ = −0.20 e Å^−^^3^
201 parameters	Extinction correction: SHELXL2014/7 (Sheldrick 2015b), Fc^*^=kFc[1+0.001xFc^2^λ^3^/sin(2θ)]^-1/4^
1 restraint	Extinction coefficient: 0.012 (3)
Primary atom site location: dual	Absolute structure: Flack x determined using 1473 quotients [(*I*^+^)-(*I*^-^)]/[(*I*^+^)+(*I*^-^)] (Parsons *et al.*, 2013)
Secondary atom site location: difference Fourier map	Absolute structure parameter: 0.4 (5)

### trans-11-Methyl-4-phenyl-1,10-dioxacyclotridec-5-ene-2,9-dione (III) Special details

**Table:**

Geometry. All esds (except the esd in the dihedral angle between two l.s. planes) are estimated using the full covariance matrix. The cell esds are taken into account individually in the estimation of esds in distances, angles and torsion angles; correlations between esds in cell parameters are only used when they are defined by crystal symmetry. An approximate (isotropic) treatment of cell esds is used for estimating esds involving l.s. planes.
Refinement. Refinement of F^2^ against ALL reflections. The weighted R-factor wR and goodness of fit S are based on F^2^, conventional R-factors R are based on F, with F set to zero for negative F^2^. The threshold expression of F^2^ > 2sigma(F^2^) is used only for calculating R-factors(gt) etc. and is not relevant to the choice of reflections for refinement. R-factors based on F^2^ are statistically about twice as large as those based on F, and R- factors based on ALL data will be even larger. The hydrogen atoms were first found in the difference map, then generated geometrically and refined as riding atoms with C-H distances = 0.95 - 0.99 angstroms and Uiso(H) = 1.2 times Ueq(C) for CH and CH2 groups and Uiso(H) = 1.5 times Ueq(C) for CH3 groups.

### trans-11-Methyl-4-phenyl-1,10-dioxacyclotridec-5-ene-2,9-dione (III) Fractional atomic coordinates and isotropic or equivalent isotropic displacement parameters (Å^2^)

**Table:**

	*x*	*y*	*z*	*U*_iso_*/*U*_eq_	
O1	0.15097 (18)	0.56820 (10)	0.5040 (3)	0.0233 (4)	
O10	0.08596 (17)	0.70330 (9)	0.6331 (3)	0.0221 (4)	
O14	0.13825 (19)	0.55047 (11)	0.1764 (4)	0.0299 (5)	
O15	0.2504 (2)	0.73924 (11)	0.7780 (4)	0.0338 (5)	
C2	0.1964 (2)	0.55372 (12)	0.3251 (5)	0.0227 (6)	
C3	0.3271 (2)	0.54399 (13)	0.3346 (5)	0.0238 (6)	
H3A	0.3477	0.5027	0.2718	0.029*	
H3B	0.3528	0.5427	0.4761	0.029*	
C4	0.3912 (2)	0.59802 (13)	0.2261 (4)	0.0221 (6)	
H4	0.3547	0.6031	0.0907	0.026*	
C5	0.3778 (2)	0.65980 (13)	0.3371 (5)	0.0217 (5)	
H5	0.4104	0.6627	0.4679	0.026*	
C6	0.3236 (2)	0.71018 (13)	0.2639 (5)	0.0228 (5)	
H6	0.2880	0.7067	0.1356	0.027*	
C7	0.3150 (3)	0.77242 (14)	0.3707 (5)	0.0249 (6)	
H7A	0.3313	0.8074	0.2751	0.030*	
H7B	0.3761	0.7740	0.4767	0.030*	
C8	0.1937 (3)	0.78283 (13)	0.4646 (5)	0.0236 (6)	
H8A	0.1850	0.8280	0.5058	0.028*	
H8B	0.1309	0.7730	0.3660	0.028*	
C9	0.1814 (3)	0.74016 (13)	0.6434 (5)	0.0231 (6)	
C11	0.0719 (2)	0.65729 (13)	0.7937 (4)	0.0227 (6)	
H11	0.1511	0.6395	0.8309	0.027*	
C12	−0.0051 (3)	0.60478 (14)	0.7118 (5)	0.0249 (6)	
H12A	−0.0009	0.5679	0.8039	0.030*	
H12B	−0.0882	0.6199	0.7106	0.030*	
C13	0.0263 (2)	0.58247 (14)	0.5055 (5)	0.0252 (6)	
H13A	−0.0200	0.5439	0.4709	0.030*	
H13B	0.0082	0.6161	0.4063	0.030*	
C15	0.5218 (2)	0.58492 (13)	0.1992 (4)	0.0215 (5)	
C16	0.5869 (2)	0.54591 (13)	0.3230 (5)	0.0238 (6)	
H16	0.5477	0.5230	0.4262	0.029*	
C17	0.7081 (3)	0.53929 (14)	0.3008 (5)	0.0261 (6)	
H17	0.7515	0.5124	0.3885	0.031*	
C18	0.7656 (2)	0.57231 (13)	0.1494 (5)	0.0258 (6)	
H18	0.8487	0.5678	0.1318	0.031*	
C19	0.7017 (3)	0.61141 (14)	0.0255 (5)	0.0260 (6)	
H19	0.7411	0.6342	−0.0778	0.031*	
C20	0.5811 (3)	0.61804 (14)	0.0487 (5)	0.0250 (6)	
H20	0.5382	0.6454	−0.0384	0.030*	
C21	0.0176 (3)	0.68939 (15)	0.9724 (5)	0.0283 (6)	
H21A	0.0712	0.7226	1.0218	0.043*	
H21B	0.0041	0.6578	1.0781	0.043*	
H21C	−0.0581	0.7087	0.9340	0.043*	

### trans-11-Methyl-4-phenyl-1,10-dioxacyclotridec-5-ene-2,9-dione (III) Atomic displacement parameters (Å^2^)

**Table:**

	*U*^11^	*U*^22^	*U*^33^	*U*^12^	*U*^13^	*U*^23^
O1	0.0202 (9)	0.0267 (10)	0.0231 (10)	0.0027 (8)	0.0020 (8)	0.0009 (8)
O10	0.0216 (9)	0.0228 (9)	0.0219 (10)	−0.0017 (7)	0.0008 (8)	0.0030 (8)
O14	0.0291 (11)	0.0330 (11)	0.0276 (11)	0.0007 (9)	−0.0002 (10)	−0.0049 (9)
O15	0.0318 (11)	0.0428 (12)	0.0268 (11)	−0.0113 (10)	−0.0055 (9)	0.0017 (10)
C2	0.0238 (13)	0.0185 (12)	0.0258 (14)	−0.0023 (10)	0.0036 (12)	−0.0009 (11)
C3	0.0218 (13)	0.0204 (12)	0.0291 (14)	0.0008 (10)	0.0050 (12)	0.0017 (11)
C4	0.0212 (12)	0.0207 (12)	0.0244 (14)	−0.0001 (10)	0.0024 (11)	0.0007 (10)
C5	0.0213 (12)	0.0223 (12)	0.0215 (12)	−0.0002 (10)	0.0023 (11)	0.0001 (10)
C6	0.0230 (12)	0.0234 (12)	0.0221 (13)	−0.0003 (10)	0.0019 (11)	−0.0012 (11)
C7	0.0247 (13)	0.0198 (12)	0.0302 (16)	0.0003 (11)	0.0055 (12)	0.0009 (11)
C8	0.0229 (13)	0.0206 (12)	0.0273 (14)	0.0020 (10)	0.0011 (11)	0.0026 (11)
C9	0.0235 (13)	0.0207 (13)	0.0249 (13)	−0.0006 (10)	0.0016 (11)	0.0002 (11)
C11	0.0219 (12)	0.0245 (13)	0.0218 (14)	0.0006 (10)	0.0012 (11)	0.0056 (11)
C12	0.0243 (13)	0.0242 (13)	0.0262 (14)	−0.0011 (11)	0.0046 (11)	0.0028 (11)
C13	0.0212 (13)	0.0274 (13)	0.0272 (15)	−0.0002 (10)	0.0034 (12)	−0.0020 (12)
C15	0.0218 (12)	0.0207 (12)	0.0221 (13)	−0.0006 (10)	0.0017 (11)	−0.0015 (10)
C16	0.0234 (13)	0.0250 (13)	0.0230 (13)	−0.0007 (10)	0.0005 (12)	0.0018 (11)
C17	0.0243 (13)	0.0255 (13)	0.0284 (15)	0.0016 (10)	0.0007 (11)	−0.0017 (11)
C18	0.0204 (12)	0.0249 (13)	0.0321 (15)	−0.0013 (10)	0.0014 (12)	−0.0060 (12)
C19	0.0259 (13)	0.0244 (13)	0.0278 (14)	−0.0030 (11)	0.0050 (12)	−0.0005 (11)
C20	0.0260 (13)	0.0226 (12)	0.0264 (14)	0.0003 (11)	0.0042 (11)	0.0009 (11)
C21	0.0313 (14)	0.0307 (14)	0.0230 (14)	0.0012 (12)	0.0039 (12)	0.0004 (12)

### trans-11-Methyl-4-phenyl-1,10-dioxacyclotridec-5-ene-2,9-dione (III) Geometric parameters (Å, º)

**Table:**

O1—C2	1.336 (4)	C11—C21	1.502 (4)
O1—C13	1.440 (3)	C11—C12	1.506 (4)
O10—C9	1.328 (3)	C11—H11	1.0000
O10—C11	1.452 (3)	C12—C13	1.498 (4)
O14—C2	1.194 (4)	C12—H12A	0.9900
O15—C9	1.191 (4)	C12—H12B	0.9900
C2—C3	1.491 (4)	C13—H13A	0.9900
C3—C4	1.527 (4)	C13—H13B	0.9900
C3—H3A	0.9900	C15—C16	1.376 (4)
C3—H3B	0.9900	C15—C20	1.394 (4)
C4—C5	1.500 (4)	C16—C17	1.383 (4)
C4—C15	1.512 (4)	C16—H16	0.9500
C4—H4	1.0000	C17—C18	1.388 (4)
C5—C6	1.315 (4)	C17—H17	0.9500
C5—H5	0.9500	C18—C19	1.370 (4)
C6—C7	1.490 (4)	C18—H18	0.9500
C6—H6	0.9500	C19—C20	1.378 (4)
C7—C8	1.523 (4)	C19—H19	0.9500
C7—H7A	0.9900	C20—H20	0.9500
C7—H7B	0.9900	C21—H21A	0.9800
C8—C9	1.499 (4)	C21—H21B	0.9800
C8—H8A	0.9900	C21—H21C	0.9800
C8—H8B	0.9900		
			
C2—O1—C13	115.4 (2)	C21—C11—C12	112.3 (2)
C9—O10—C11	115.9 (2)	O10—C11—H11	109.5
O14—C2—O1	123.2 (3)	C21—C11—H11	109.5
O14—C2—C3	124.9 (3)	C12—C11—H11	109.5
O1—C2—C3	111.9 (3)	C13—C12—C11	115.2 (2)
C2—C3—C4	110.4 (2)	C13—C12—H12A	108.5
C2—C3—H3A	109.6	C11—C12—H12A	108.5
C4—C3—H3A	109.6	C13—C12—H12B	108.5
C2—C3—H3B	109.6	C11—C12—H12B	108.5
C4—C3—H3B	109.6	H12A—C12—H12B	107.5
H3A—C3—H3B	108.1	O1—C13—C12	107.6 (2)
C5—C4—C15	108.3 (2)	O1—C13—H13A	110.2
C5—C4—C3	110.9 (2)	C12—C13—H13A	110.2
C15—C4—C3	112.6 (2)	O1—C13—H13B	110.2
C5—C4—H4	108.3	C12—C13—H13B	110.2
C15—C4—H4	108.3	H13A—C13—H13B	108.5
C3—C4—H4	108.3	C16—C15—C20	118.2 (3)
C6—C5—C4	123.8 (3)	C16—C15—C4	123.9 (3)
C6—C5—H5	118.1	C20—C15—C4	117.7 (2)
C4—C5—H5	118.1	C15—C16—C17	121.6 (3)
C5—C6—C7	123.7 (3)	C15—C16—H16	119.2
C5—C6—H6	118.1	C17—C16—H16	119.2
C7—C6—H6	118.1	C16—C17—C18	119.4 (3)
C6—C7—C8	112.5 (2)	C16—C17—H17	120.3
C6—C7—H7A	109.1	C18—C17—H17	120.3
C8—C7—H7A	109.1	C19—C18—C17	119.5 (3)
C6—C7—H7B	109.1	C19—C18—H18	120.2
C8—C7—H7B	109.1	C17—C18—H18	120.2
H7A—C7—H7B	107.8	C18—C19—C20	120.8 (3)
C9—C8—C7	109.1 (2)	C18—C19—H19	119.6
C9—C8—H8A	109.9	C20—C19—H19	119.6
C7—C8—H8A	109.9	C19—C20—C15	120.4 (3)
C9—C8—H8B	109.9	C19—C20—H20	119.8
C7—C8—H8B	109.9	C15—C20—H20	119.8
H8A—C8—H8B	108.3	C11—C21—H21A	109.5
O15—C9—O10	124.1 (3)	C11—C21—H21B	109.5
O15—C9—C8	123.5 (3)	H21A—C21—H21B	109.5
O10—C9—C8	112.4 (3)	C11—C21—H21C	109.5
O10—C11—C21	109.6 (2)	H21A—C21—H21C	109.5
O10—C11—C12	106.2 (2)	H21B—C21—H21C	109.5
			
C13—O1—C2—O14	−2.5 (4)	O10—C11—C12—C13	44.7 (3)
C13—O1—C2—C3	176.1 (2)	C21—C11—C12—C13	164.5 (2)
O14—C2—C3—C4	67.8 (4)	C2—O1—C13—C12	−173.5 (2)
O1—C2—C3—C4	−110.8 (3)	C11—C12—C13—O1	51.2 (3)
C2—C3—C4—C5	68.5 (3)	C5—C4—C15—C16	96.9 (3)
C2—C3—C4—C15	−169.9 (2)	C3—C4—C15—C16	−26.2 (4)
C15—C4—C5—C6	118.9 (3)	C5—C4—C15—C20	−78.3 (3)
C3—C4—C5—C6	−117.0 (3)	C3—C4—C15—C20	158.6 (3)
C4—C5—C6—C7	−177.2 (3)	C20—C15—C16—C17	0.1 (4)
C5—C6—C7—C8	−102.7 (3)	C4—C15—C16—C17	−175.1 (3)
C6—C7—C8—C9	72.7 (3)	C15—C16—C17—C18	−0.5 (4)
C11—O10—C9—O15	−3.5 (4)	C16—C17—C18—C19	0.6 (4)
C11—O10—C9—C8	175.8 (2)	C17—C18—C19—C20	−0.4 (5)
C7—C8—C9—O15	54.4 (4)	C18—C19—C20—C15	−0.1 (5)
C7—C8—C9—O10	−125.0 (3)	C16—C15—C20—C19	0.2 (4)
C9—O10—C11—C21	82.1 (3)	C4—C15—C20—C19	175.6 (3)
C9—O10—C11—C12	−156.3 (2)		
