# Supplementary material for: Structures of three disubstituted [13]-macro­di­lac­tones reveal effects of substitution on macrocycle conformation
Source: Acta Crystallogr E Crystallogr Commun. 2020 Sep 8;76(Pt 10):1617–23. doi: 10.1107/S2056989020012037 (PMC7534246; doi:10.1107/S2056989020012037)
Supplement: Supplementary file 5 [file e-76-01617-sup5.pdf]

Supporting Information for:

Structures of three di-substituted [13]-macrodilactones reveal effects of substitution on macrocycle conformation

Kelli M. Rutledge[a], Caleb Griesbach[a], Brandon Q. Mercado[b]*, Mark W. Peczuh[a]*
[a]*Department of Chemistry, University of Connecticut, 55 N. Eagleville Road, U3060, Storrs, CT 06269, USA*
[b]*Department of Chemistry, Yale University, PO Box 208107, New Haven, Connecticut 06520-8107, USA*

Total number of pages: 26

Table of Contents

	Item	Page(s)
	**Figure S1**. The fragments used to search the Cambridge Structure Database (CSD 2017 + 4 updates). The dashed lines highlight the 3D contacts defined within ConQuest.	2
	**Figure S2**. Close contact in compound **I**	2
	**Figure S3.** The distances (DIST1 in Å) for 3796 structures with intermolecular phenyl bromine interactions (CSD 2017 + 4 updates).	3
	**Figure S4**. Close contact in compound **II**	3
	**Figure S5.** The distances (DIST1 in Å) for 2486 structures with intermolecular phenyl bromine interactions (CSD 2017 + 4 updates).	4
	**Figure S6**. Close contact in compound **III**	4
	**Figure S7.** The distances (DIST1 in Å) for 88 structures with intermolecular phenyl bromine interactions (CSD 2017 + 4 updates).	5
	**Table S1.** Substitution patterns and refcodes for [13]-macrodilactones	6
	**Table S2.** Aspect ratios of compounds in Figure 3 from X-ray crystallographic data	7
	**Table S3.** RMSD (Å) values comparing X-ray and DFT optimized structures for the core ring atoms of the [13]-macrodilactones	8
	**Table S4.** Torsion angles, bond angles, and bond lengths of compound "*a*" ring atoms.	9-10
	**Table S5.** Torsion angles, bond angles, and bond lengths of compound "*g*" **II** ring atoms.	10-11
	**Table S6.** Torsion angles, bond angles, and bond lengths of compound "*e*" ring atoms.	11-12
	**Table S7.** Torsion angles, bond angles, and bond lengths of compound "*h*" **I** ring atoms.	12-13
	**Table S8.** Torsion angles, bond angles, and bond lengths of compound "*k*" **III** ring atoms.	13-14
	^{1}H and ^{13}C spectra for new compounds	15-26

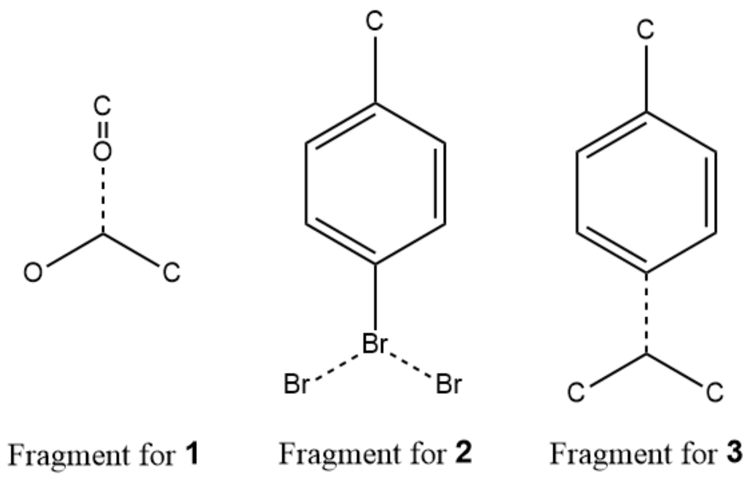

Figure S1. The fragments used to search the Cambridge Structure Database (CSD 2017 + 4 updates). The dashed lines highlight the 3D contacts defined within ConQuest.

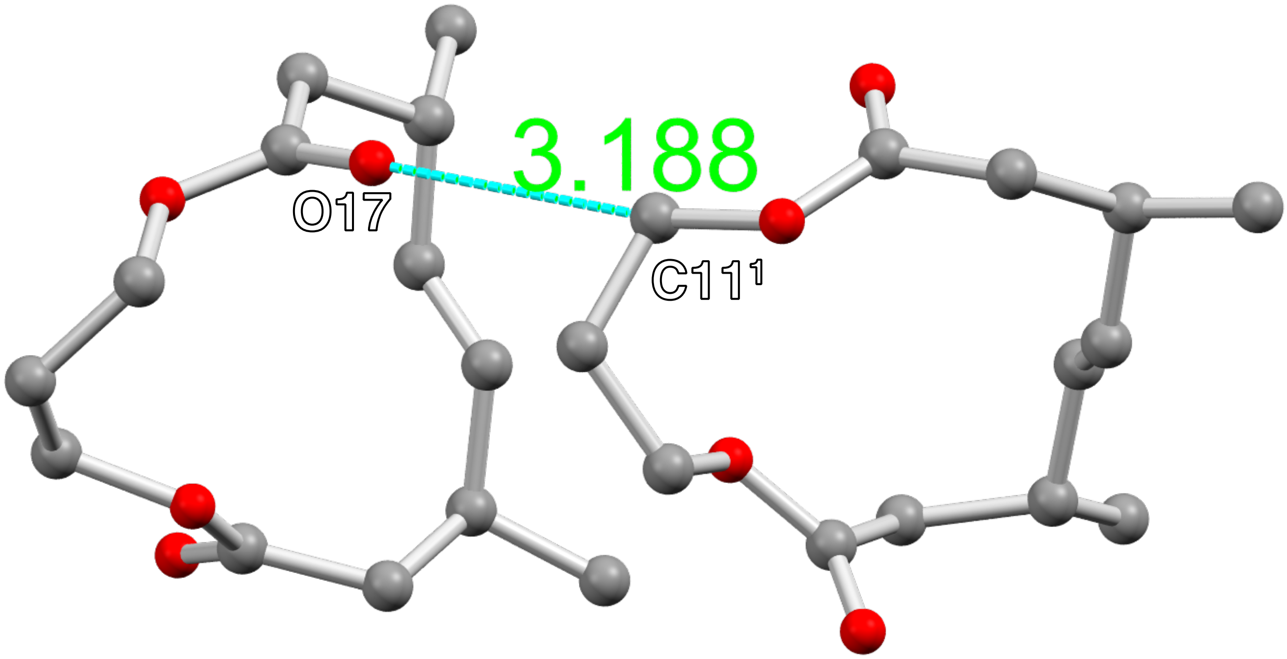

Figure S2. Interaction identified in **I**. Operator 1 is $x, \frac{3}{2} - y, -\frac{1}{2} + z$.

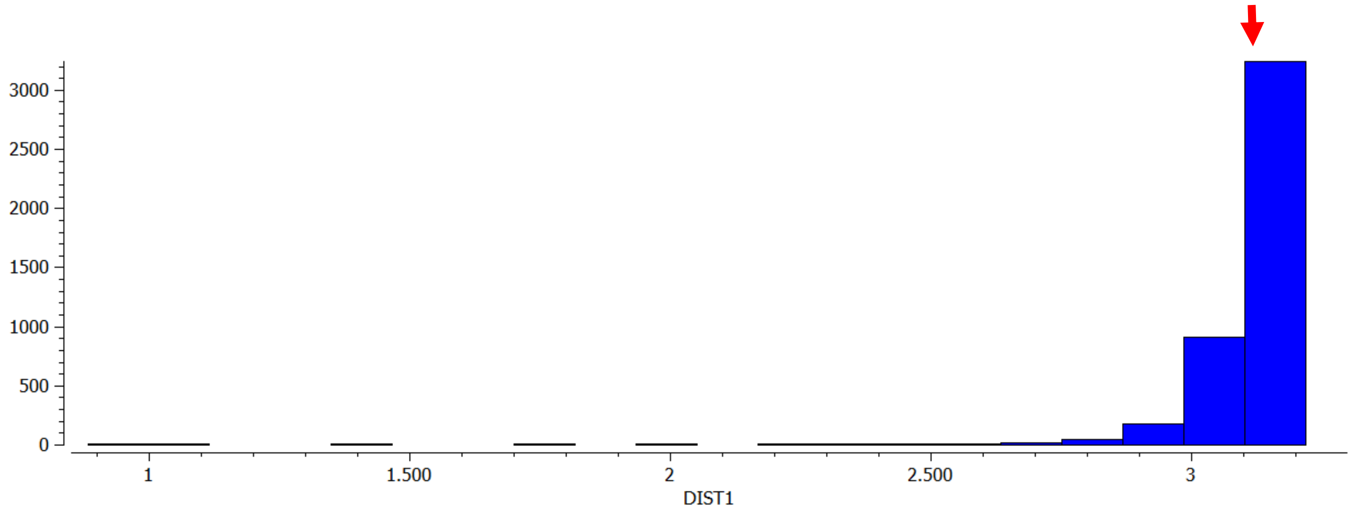

Figure S3. The distances (DIST1 in Å) for 3796 structures when searching for fragment 1 (CSD 2017 + 4 updates). The red arrow identifies were the distance identified in **S2** falls in the histogram.

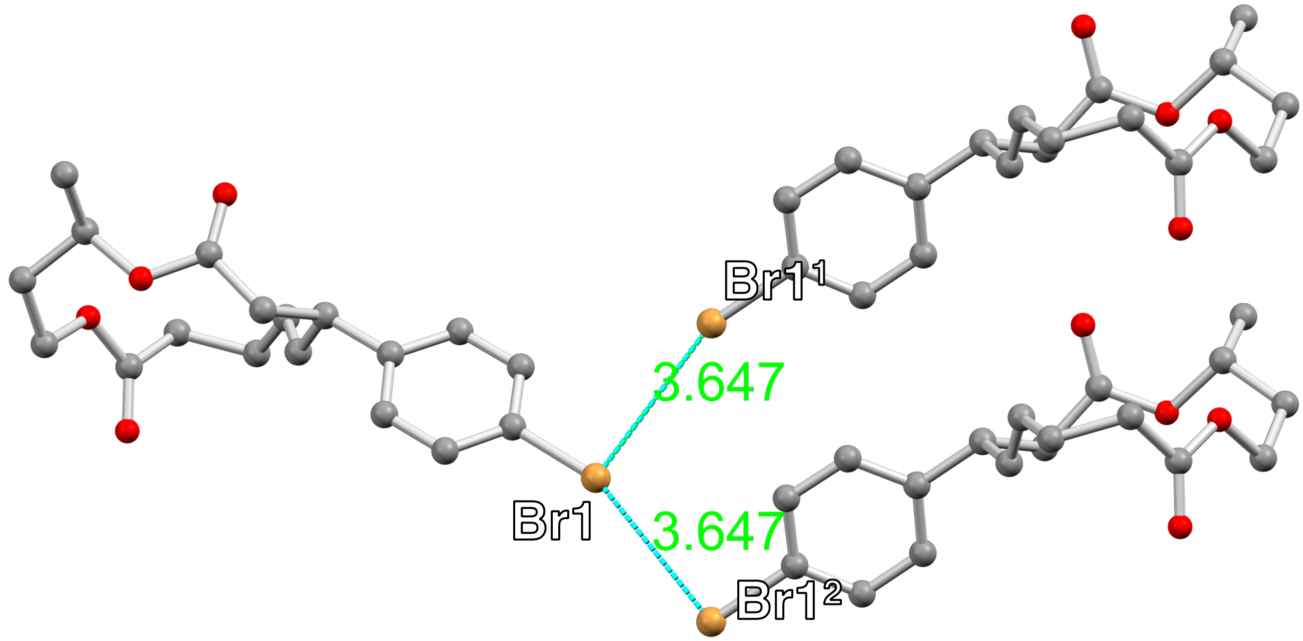

Figure S4. Interaction identified in **II**. Operator 1 is $\frac{3}{2}-x,\frac{1}{2}+y,\frac{1}{2}-z$; operator 2 is $\frac{3}{2}-x,-\frac{1}{2}+y,\frac{1}{2}-z$

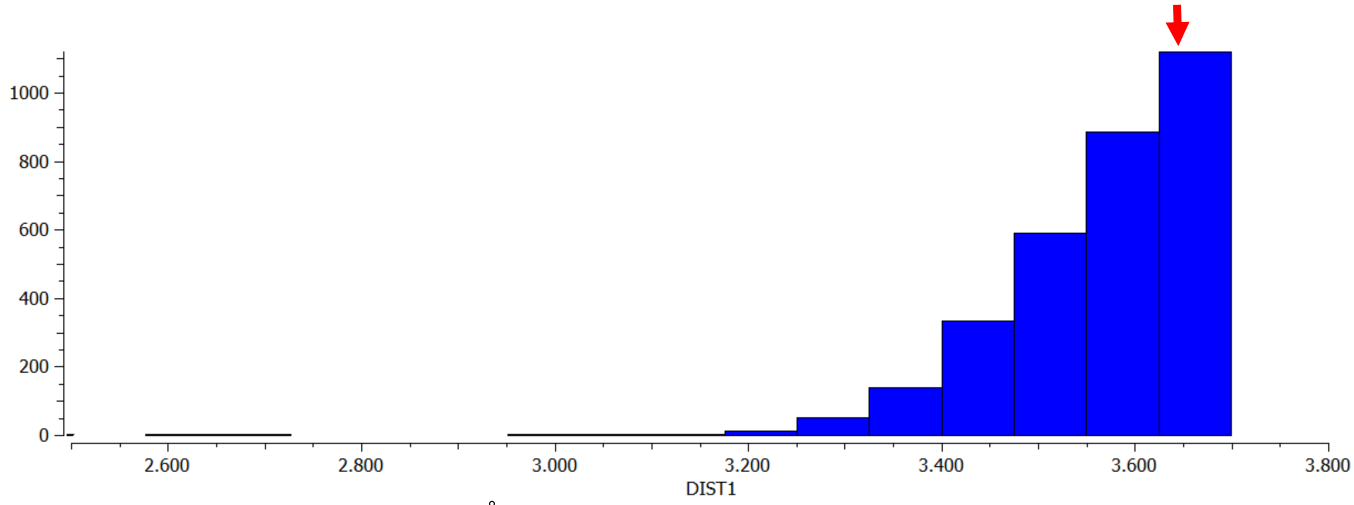

Figure S5. The distances (DIST1 in Å) for 2486 structures when searching for fragment 2 interactions (CSD 2017 + 4 updates). The red arrow identifies were the distance identified in **S4** falls in the histogram.

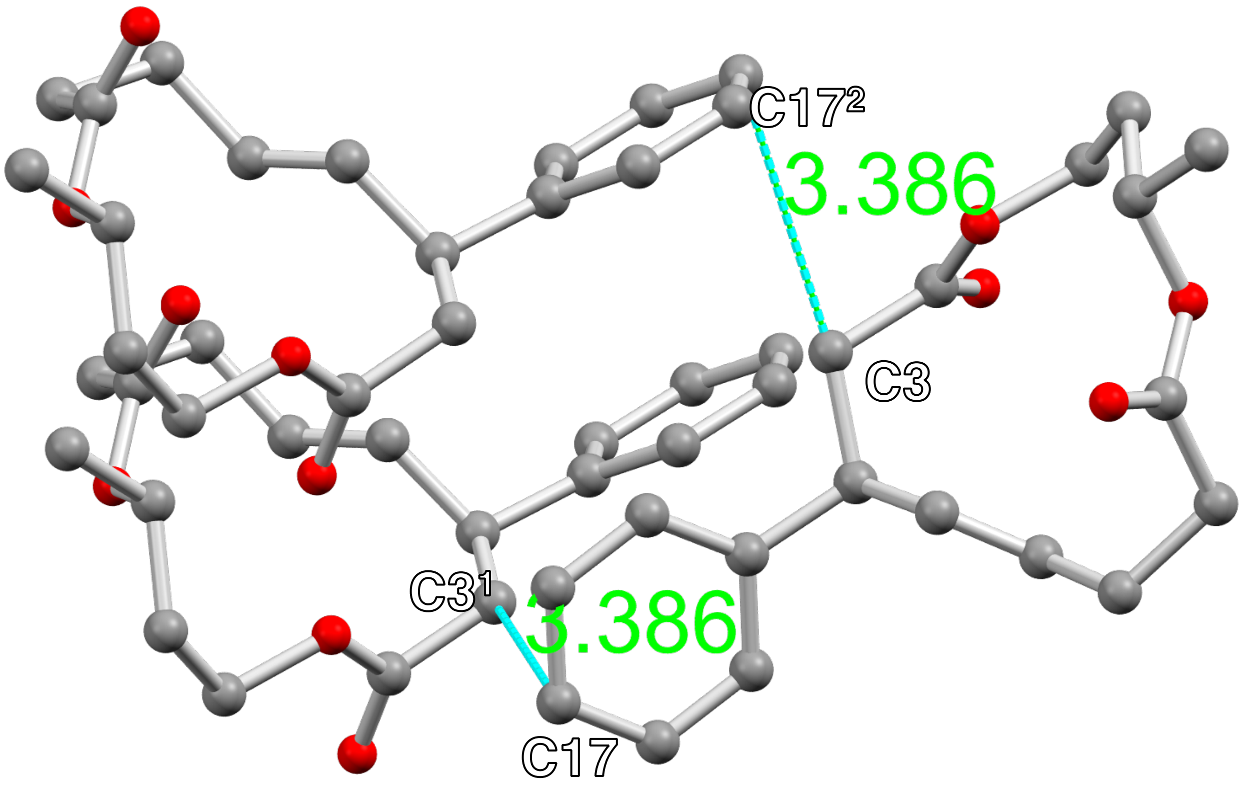

Figure S6. Interaction identified in **III**. Operator 1 is $1-x, 1-y, -\frac{1}{2}+z$; operator 2 is $1-x, 1-y, \frac{1}{2}+z$

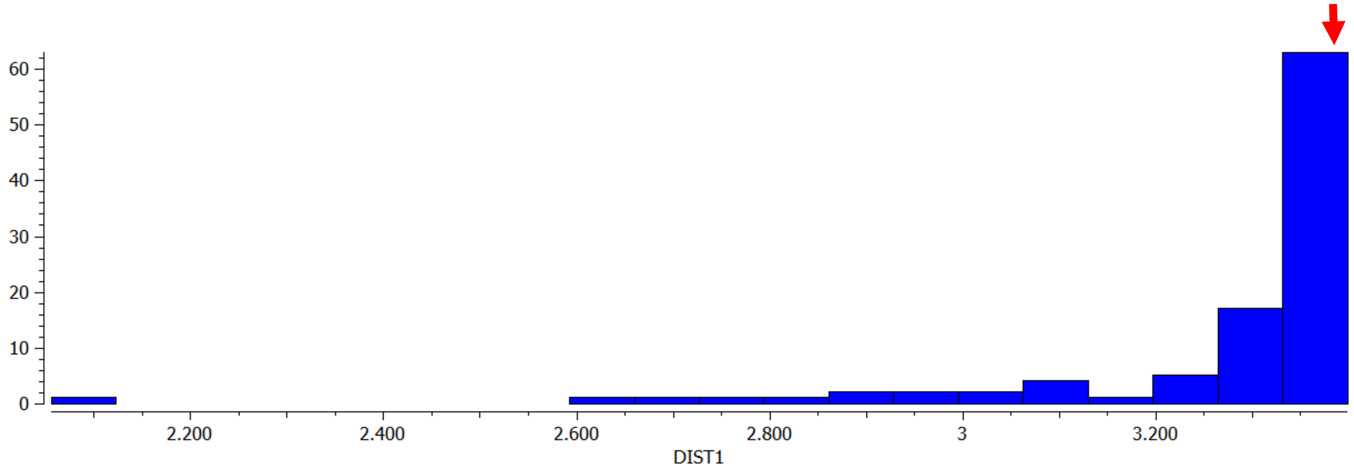

Figure S7. The distances (DIST1 in Å) for 88 structures when searching for fragment 3 interactions (CSD 2017 + 4 updates). The red arrow identifies were the distance identified in **S6** falls in the histogram.

Table S1. Substitution patterns and refcodes for [13]-macrodilactones

Entry	Fig X	Conf.	Subs.*	*cis/trans*	Code	Citation	Comments
1	a	ribbon	11,13	*trans*	URILEO	Ma & Peczuh, 2013	
2	b	ribbon	11 (mono)	-	KOHLAV	Fyvie & Peczuh, 2008a	
3	c	ribbon	3 (mono)	-	XUFKOA	Magpusao, Rutledge et al., 2015	Z'=2
4	-	ribbon	D-gluco	*trans*	XOCWIW	Fyvie & Peczuh, 2008b	
5	d	ribbon	3,8	*trans*	XUFLAN	Magpusao, Rutledge et al., 2016	
6	e	ribbon	-	-	IJEHAI	Magpusao, Rutledge et al., 2016	
7	f	ribbon	3,11	*cis*	IJEHOW	Magpusao, Rutledge et al., 2016	
**8**	g	ribbon	4,13	*cis*	**II**	This work	CCDC 1944827, 007-16012
**9**	h	ribbon	4,8	*trans*	**I**	This work	CCDC 1944826, Mini-15062
10	i	ribbon	4 (mono)	-	ECOYED	Rutledge, Hamlin et al., 2017	
11	j	ribbon	3,13	*trans*	IJEHEM	Magpusao, Rutledge et al., 2016	
10'	-	other	4 (mono)	-	ECOYED		
12	-	other	11,13	*cis*	URILAK	Ma & Peczuh, 2013	
3'	c'	other	3 (mono)	-	XUFKOA		Z'=2
13	-	other	11,13	*cis*	URILAK	Ma & Peczuh, 2013	
14	-	other	3,11	*trans*	IJEHUC	Magpusao, Rutledge *et al.*, 2016	
**15**	k	ribbon	4,11	*trans*	**III**	This work	CCDC 1944828, Mini-16062
16	-	other	3,8	*cis*	XUFKUG	Magpusao, Rutledge *et al.*, 2016	
17	-	other	8,11	*cis*	IJEHIQ	Magpusao, Rutledge *et al.*, 2016	

* Subs. = positions on the [13]-macrodilactone that are substituted.

Fyvie, W.S. & Peczuh, M.W. (2008a). *Chem. Commun.* 4028-4030.

Fyvie, W.S. & Peczuh, M.W. (2008b). *J. Org. Chem.* **73**, 3626-3629.

Ma, J. & Peczuh, M. W. (2013). *J. Org. Chem.* **78**, 7414-7422.

Magpusao, A.N., Rutledge, K., Mercado, B., & Peczuh, M.W. (2015) *Org. Biomol. Chem.* 13, 5086-5089.

Magpusao, A.N., Rutledge, K.M., Hamlin, T.A., Lawrence, J.-M., Mercado, B.Q., Leadbeater, N.E. & Peczuh, M.W. (2016). *Chem. Eur. J.* **22**, 6001-6011.

Rutledge, K.M., Hamlin, T.A., Baldisseri, D., & Peczuh, M.W. (2017). *Chem. Asian J.* **12**, 2623-2633.

Table S2. Aspect ratios of compounds in Figure 3 from X-ray crystallographic data

code	cmpd	length	width	aspect ratio
URILEO	*a*	5.3989(12)	4.0809(13)	1.323
KOHLAV	*b*	5.394(3)	4.073(4)	1.32
XUFKOA	*c*	5.3893(18)	4.108(2)	1.31
XOCWIW	-	5.387(3)	4.102(3)	1.31
XUFLAN	*d*	5.370(4)	4.098(3)	1.31
IJEHAI	*e*	5.3663(11)	4.1487(13)	1.293
IJEHOW	*f*	5.3621(15)	4.1684(16)	1.286
**II**	*g*	5.327(2)	4.272(3)	1.25
**I**	*h*	5.327(3)	4.231(4)	1.26
ECOYED	*i*	5.282(5)	4.383(4)	1.21
IJEHEM	*j*	5.2478(18)	4.365(2)	1.20
ECOYED	-	5.247(5)	4.383(4)	
URILAK	-	5.233(4)	4.391(4)	
XUFKOA	*c'*	5.230(2)	4.3762(19)	1.20
URILAX	-	5.207(9)	4.391(4)	
IJEHUC	-	5.192(2)	4.245(18)	
**III**	*k*	5.151(3)	4.453(4)	1.16
XUFKUG	-	4.780(2)	4.4378(15)	
IJEHIQ	-	4.682(1)	4.5502(15)	

Notes:
length = C12 to alkene centroid distance (Å)
width = C2 to C9 (carbonyl carbons) distance (Å)
aspect ratio = length/width

Table S3. RMSD (Å) values comparing X-ray and DFT optimized structures for the core ring atoms of the [13]-macrodilactones

**Compound**	**Functional/Basis Set**				
	B3LYD-D3/ CC-PVDC	*M06-2X-D3/ CC-PVDC*	*B3LYD-D3/ LACVP***	*M06-2X-D3/ LACVP***	*average*
a (URILEO) C2/c	0.048	0.070	0.046	0.057	0.055
e (IJEHAI) $P2_1/c$	0.061	0.076	0.113	0.102	0.088
g (**II**) $P2_1/c$	0.122	0.202	0.194	0.120	0.160
h (**I**) $P2_1/n$	0.103	0.119	0.096	0.094	0.103
k (**III**) $Pna2_1$	0.288	0.251	0.265	0.200	0.251

Table S4. Torsion angles, bond angles, and bond lengths of compound "*a*" ring atoms.

**Torsion Angles**					
	A	B	C	D	XRD
C(2)-O(1)-C(13)-C(12)	149.75	149.07	151.12	150.91	151.42
O(1)-C(13)-C(12)-C(11)	51.01	50.75	51.05	50.58	49.97
C(13)-C(12)-C(11)-O(10)	51.08	50.75	51.06	50.51	49.89
C(12)-C(11)-O(10)-C(9)	149.57	149.08	151.06	150.94	151.35
C(11)-O(10)-C(9)-C(8)	174.67	176.14	175.11	176.84	176.59
O(10)-C(9)-C(8)-C(7)	139.79	142.18	137.63	139.33	135.32
C(9)-C(8)-C(7)-C(6)	68.24	68.10	66.52	66.62	67.06
C(8)-C(7)-C(6)-C(5)	102.10	102.12	101.53	102.09	103.24
C(7)-C(6)-C(5)-C(4)	171.50	170.91	173.90	173.68	175.27
C(6)-C(5)-C(4)-C(3)	102.04	102.08	101.75	102.13	103.21
C(5)-C(4)-C(3)-C(2)	68.35	68.06	66.49	66.49	67.05
C(4)-C(3)-C(2)-O(1)	139.67	142.17	137.50	139.30	135.30
C(3)-C(2)-O(1)-C(13)	174.65	176.17	175.12	176.90	176.58
**Bond Angles**					
	A	B	C	D	XRD
C(2)-O(1)-C(13)	116.51	116.44	116.56	116.39	116.04
O(1)-C(13)-C(12)	106.62	106.42	106.40	106.24	107.16
C(13)-C(12)-C(11)	114.03	112.87	113.55	112.56	114.90
C(12)-C(11)-O(10)	106.62	106.41	106.41	106.23	107.24
C(11)-O(10)-C(9)	116.51	116.44	116.55	116.39	116.05
O(10)-C(9)-C(8)	112.31	112.42	111.89	112.08	111.96
C(9)-C(8)-C(7)	110.57	110.12	110.20	109.72	110.48
C(8)-C(7)-C(6)	111.09	110.34	111.40	110.61	111.20
C(7)-C(6)-C(5)	124.92	124.52	124.45	124.06	124.73
C(6)-C(5)-C(4)	124.93	124.51	124.45	124.05	124.75
C(5)-C(4)-C(3)	111.10	110.34	111.41	110.62	111.18
C(4)-C(3)-C(2)	110.57	110.14	110.20	109.73	110.53
C(3)-C(2)-O(1)	112.31	112.43	111.90	112.08	111.99
**Bond Lengths (Å)**					
	A	B	C	D	XRD
O(1)-C(13)	1.46	1.44	1.46	1.44	1.47
C(13)-C(12)	1.52	1.52	1.53	1.52	1.52
C(12)-C(11)	1.52	1.52	1.53	1.52	1.52
C(11)-O(10)	1.46	1.44	1.46	1.44	1.47
O(10)-C(9)	1.35	1.34	1.35	1.34	1.35
C(9)-C(8)	1.51	1.50	1.52	1.51	1.51
C(8)-C(7)	1.54	1.53	1.54	1.54	1.53
C(7)-C(6)	1.50	1.50	1.51	1.50	1.50
C(6)-C(5)	1.33	1.33	1.34	1.33	1.33
C(5)-C(4)	1.50	1.50	1.51	1.50	1.50
C(4)-C(3)	1.54	1.53	1.54	1.54	1.53

C(3)-C(2)	1.51	1.50	1.52	1.51	1.51
C(2)-O(1)	1.35	1.34	1.35	1.34	1.35

A = B3LYP-D3/cc-PVDC
B = M06-2X-D3/cc-PVDC
C = B3LYP-D3/LACVP**
D = M06-2X-D3/LACVP**

Table S5. Torsion angles, bond angles, and bond lengths of compound "*g*" (**II**) ring atoms.

**Torsion Angles**					
	A	B	C	D	XRD
C(2)-O(1)-C(13)-C(12)	149.63	149.90	150.79	151.03	155.42
O(1)-C(13)-C(12)-C(11)	49.61	49.76	50.33	49.90	50.10
C(13)-C(12)-C(11)-O(10)	52.09	50.87	52.55	51.98	49.63
C(12)-C(11)-O(10)-C(9)	148.03	142.56	150.06	149.59	161.76
C(11)-O(10)-C(9)-C(8)	174.74	175.80	174.07	176.19	174.91
O(10)-C(9)-C(8)-C(7)	141.28	148.04	138.64	140.44	125.30
C(9)-C(8)-C(7)-C(6)	67.97	67.99	65.61	65.74	70.30
C(8)-C(7)-C(6)-C(5)	102.03	102.05	99.99	101.74	105.86
C(7)-C(6)-C(5)-C(4)	171.51	169.43	175.23	174.15	177.36
C(6)-C(5)-C(4)-C(3)	103.55	102.12	102.41	103.05	107.00
C(5)-C(4)-C(3)-C(2)	70.28	68.88	65.58	68.14	71.58
C(4)-C(3)-C(2)-O(1)	141.37	144.57	137.99	140.69	132.10
C(3)-C(2)-O(1)-C(13)	175.88	179.16	175.78	176.63	174.11
**Bond Angles**					
	A	B	C	D	XRD
C(2)-O(1)-C(13)	116.42	116.17	116.80	116.50	116.30
O(1)-C(13)-C(12)	106.78	106.79	106.31	106.18	105.89
C(13)-C(12)-C(11)	113.43	112.41	112.90	111.94	113.93
C(12)-C(11)-O(10)	108.36	108.12	107.81	107.53	107.42
C(11)-O(10)-C(9)	116.54	116.73	116.73	116.50	115.77
O(10)-C(9)-C(8)	112.10	112.04	111.60	111.90	111.61
C(9)-C(8)-C(7)	110.59	110.44	110.15	109.74	110.55
C(8)-C(7)-C(6)	110.96	110.06	111.47	110.49	111.75
C(7)-C(6)-C(5)	124.96	124.63	124.03	123.80	124.21
C(6)-C(5)-C(4)	124.88	124.27	124.66	124.20	124.33
C(5)-C(4)-C(3)	109.20	108.38	110.25	109.33	109.79
C(4)-C(3)-C(2)	110.46	110.17	110.42	109.43	109.71
C(3)-C(2)-O(1)	112.29	112.54	111.56	111.77	112.20
**Bond Lengths (Å)**					
	A	B	C	D	XRD
O(1)-C(13)	1.46	1.45	1.46	1.44	1.47
C(13)-C(12)	1.52	1.52	1.53	1.52	1.52
C(12)-C(11)	1.51	1.51	1.52	1.52	1.51
C(11)-O(10)	1.45	1.43	1.45	1.43	1.45

O(10)-C(9)	1.35	1.34	1.35	1.34	1.34
C(9)-C(8)	1.51	1.50	1.52	1.51	1.51
C(8)-C(7)	1.53	1.53	1.54	1.54	1.54
C(7)-C(6)	1.50	1.50	1.51	1.50	1.50
C(6)-C(5)	1.33	1.33	1.34	1.33	1.33
C(5)-C(4)	1.51	1.50	1.51	1.51	1.51
C(4)-C(3)	1.54	1.53	1.55	1.54	1.55
C(3)-C(2)	1.51	1.50	1.52	1.51	1.51
C(2)-O(1)	1.35	1.34	1.35	1.34	1.34

A = B3LYP-D3/cc-PVDC
B = M06-2X-D3/cc-PVDC
C = B3LYP-D3/LACVP**
D = M06-2X-D3/LACVP**

Table S6. Torsion angles, bond angles, and bond lengths of compound “*e*” ring atoms.

**Torsion Angles**					
	A	B	C	D	XRD
C(2)-O(1)-C(13)-C(12)	156.67	158.96	153.30	151.10	168.10
O(1)-C(13)-C(12)-C(11)	51.95	51.86	52.14	51.53	52.76
C(13)-C(12)-C(11)-O(10)	51.56	50.79	51.85	51.45	48.62
C(12)-C(11)-O(10)-C(9)	145.37	139.08	149.48	149.56	141.96
C(11)-O(10)-C(9)-C(8)	173.78	174.55	173.94	175.96	172.56
O(10)-C(9)-C(8)-C(7)	140.99	147.55	137.94	140.34	142.12
C(9)-C(8)-C(7)-C(6)	67.65	67.05	66.53	66.40	67.65
C(8)-C(7)-C(6)-C(5)	102.24	102.43	101.92	102.16	101.74
C(7)-C(6)-C(5)-C(4)	172.93	171.51	174.47	173.73	175.46
C(6)-C(5)-C(4)-C(3)	103.77	102.69	102.09	102.09	103.93
C(5)-C(4)-C(3)-C(2)	69.16	68.81	66.84	66.60	66.45
C(4)-C(3)-C(2)-O(1)	133.64	135.48	135.67	139.46	122.45
C(3)-C(2)-O(1)-C(13)	174.30	176.82	174.31	176.11	176.69
**Bond Angles**					
	A	B	C	D	XRD
C(2)-O(1)-C(13)	116.08	115.72	116.38	116.35	115.65
O(1)-C(13)-C(12)	108.10	107.88	107.80	107.54	107.44
C(13)-C(12)-C(11)	112.71	111.78	112.29	111.26	112.90
C(12)-C(11)-O(10)	108.22	108.08	107.81	107.55	107.17
C(11)-O(10)-C(9)	116.70	116.90	116.65	116.46	117.04
O(10)-C(9)-C(8)	111.99	111.90	111.67	111.90	111.39
C(9)-C(8)-C(7)	110.55	110.41	110.24	109.74	110.59
C(8)-C(7)-C(6)	111.43	110.71	111.62	110.64	111.20
C(7)-C(6)-C(5)	124.75	124.18	124.37	124.01	125.29
C(6)-C(5)-C(4)	125.18	124.87	124.52	124.07	124.52
C(5)-C(4)-C(3)	111.30	110.19	111.50	110.60	112.54
C(4)-C(3)-C(2)	110.22	109.63	110.11	109.66	110.40

C(3)-C(2)-O(1)	112.44	112.72	111.83	111.97	112.40
**Bond Lengths (Å)**					
	A	B	C	D	XRD
O(1)-C(13)	1.44	1.43	1.45	1.43	1.46
C(13)-C(12)	1.52	1.51	1.52	1.52	1.52
C(12)-C(11)	1.52	1.51	1.52	1.52	1.52
C(11)-O(10)	1.45	1.43	1.45	1.43	1.45
O(10)-C(9)	1.35	1.35	1.35	1.34	1.35
C(9)-C(8)	1.51	1.51	1.52	1.51	1.51
C(8)-C(7)	1.54	1.53	1.54	1.54	1.54
C(7)-C(6)	1.50	1.50	1.51	1.50	1.50
C(6)-C(5)	1.33	1.33	1.34	1.33	1.32
C(5)-C(4)	1.50	1.50	1.51	1.50	1.51
C(4)-C(3)	1.54	1.53	1.55	1.54	1.54
C(3)-C(2)	1.51	1.50	1.52	1.51	1.51
C(2)-O(1)	1.35	1.34	1.35	1.34	1.35

A = B3LYP-D3/cc-PVDC
B = M06-2X-D3/cc-PVDC
C = B3LYP-D3/LACVP**
D = M06-2X-D3/LACVP**

Table S7. Torsion angles, bond angles, and bond lengths of compound “*h*” **I** ring atoms.

**Torsion Angles**					
	A	B	C	D	XRD
C(2)-O(1)-C(13)-C(12)	150.99	148.95	149.91	148.44	151.07
O(1)-C(13)-C(12)-C(11)	51.49	51.12	51.70	51.20	49.64
C(13)-C(12)-C(11)-O(10)	51.52	51.22	51.95	51.66	50.46
C(12)-C(11)-O(10)-C(9)	149.95	149.11	153.66	155.57	166.35
C(11)-O(10)-C(9)-C(8)	174.65	176.37	174.62	176.45	173.06
O(10)-C(9)-C(8)-C(7)	138.59	141.69	135.27	135.44	125.79
C(9)-C(8)-C(7)-C(6)	66.88	66.58	65.94	65.73	73.62
C(8)-C(7)-C(6)-C(5)	102.68	102.31	102.60	102.64	101.60
C(7)-C(6)-C(5)-C(4)	175.47	174.06	177.43	177.34	177.42
C(6)-C(5)-C(4)-C(3)	102.17	101.85	101.81	102.37	109.64
C(5)-C(4)-C(3)-C(2)	67.23	66.79	65.80	65.50	66.32
C(4)-C(3)-C(2)-O(1)	138.19	142.01	137.78	139.80	132.19
C(3)-C(2)-O(1)-C(13)	174.58	176.21	174.15	175.35	174.67
**Bond Angles**					
	A	B	C	D	XRD
C(2)-O(1)-C(13)	116.45	116.42	116.60	116.55	116.52
O(1)-C(13)-C(12)	108.09	107.86	107.76	107.47	107.50
C(13)-C(12)-C(11)	112.57	111.55	112.20	111.20	112.67
C(12)-C(11)-O(10)	108.09	107.85	107.75	107.44	107.50
C(11)-O(10)-C(9)	116.55	116.43	116.38	116.06	115.27

O(10)-C(9)-C(8)	112.10	112.26	111.86	112.17	112.89
C(9)-C(8)-C(7)	111.15	110.78	110.63	110.08	111.52
C(8)-C(7)-C(6)	109.88	109.19	110.17	109.61	109.75
C(7)-C(6)-C(5)	125.32	124.93	125.02	124.76	125.22
C(6)-C(5)-C(4)	125.42	124.99	124.93	124.51	126.13
C(5)-C(4)-C(3)	109.84	109.18	110.23	109.78	110.36
C(4)-C(3)-C(2)	111.13	110.78	110.78	110.37	112.29
C(3)-C(2)-O(1)	112.16	112.27	111.72	111.88	112.05
**Bond Lengths (Å)**					
	A	B	C	D	XRD
O(1)-C(13)	1.44	1.43	1.45	1.43	1.45
C(13)-C(12)	1.52	1.51	1.52	1.52	1.51
C(12)-C(11)	1.52	1.51	1.52	1.52	1.50
C(11)-O(10)	1.44	1.43	1.45	1.43	1.45
O(10)-C(9)	1.35	1.34	1.35	1.34	1.33
C(9)-C(8)	1.51	1.50	1.52	1.51	1.50
C(8)-C(7)	1.54	1.53	1.55	1.54	1.53
C(7)-C(6)	1.50	1.50	1.51	1.51	1.51
C(6)-C(5)	1.33	1.33	1.34	1.33	1.30
C(5)-C(4)	1.50	1.50	1.51	1.51	1.50
C(4)-C(3)	1.54	1.53	1.55	1.54	1.52
C(3)-C(2)	1.51	1.50	1.52	1.51	1.49
C(2)-O(1)	1.35	1.34	1.35	1.34	1.34

A = B3LYP-D3/cc-PVDC
B = M06-2X-D3/cc-PVDC
C = B3LYP-D3/LACVP**
D = M06-2X-D3/LACVP**

Table S8. Torsion angles, bond angles, and bond lengths of compound "*k*" **III** ring atoms.

**Torsion Angles**					
	A	B	C	D	XRD
C(2)-O(1)-C(13)-C(12)	146.27	148.07	149.18	150.05	156.26
O(1)-C(13)-C(12)-C(11)	49.66	49.20	50.75	50.35	44.67
C(13)-C(12)-C(11)-O(10)	52.26	51.79	52.60	52.06	51.24
C(12)-C(11)-O(10)-C(9)	150.26	147.45	153.50	153.34	173.45
C(11)-O(10)-C(9)-C(8)	175.43	177.30	174.31	176.11	176.09
O(10)-C(9)-C(8)-C(7)	141.07	144.58	135.55	136.94	110.82
C(9)-C(8)-C(7)-C(6)	67.89	67.44	65.07	65.49	68.45
C(8)-C(7)-C(6)-C(5)	103.12	103.72	103.53	104.97	117.02
C(7)-C(6)-C(5)-C(4)	171.22	170.71	175.93	176.14	177.15
C(6)-C(5)-C(4)-C(3)	101.02	101.28	99.77	100.31	102.70
C(5)-C(4)-C(3)-C(2)	68.92	69.36	66.31	66.64	72.75
C(4)-C(3)-C(2)-O(1)	143.01	143.80	138.50	139.18	125.06
C(3)-C(2)-O(1)-C(13)	175.31	177.06	174.93	176.38	175.80

Bond Angles					
	A	B	C	D	XRD
C(2)-O(1)-C(13)	116.57	116.30	116.61	116.40	115.95
O(1)-C(13)-C(12)	106.77	106.63	106.35	106.24	106.29
C(13)-C(12)-C(11)	113.23	112.28	112.77	111.87	115.19
C(12)-C(11)-O(10)	108.24	108.06	107.66	107.37	107.63
C(11)-O(10)-C(9)	116.49	116.48	116.55	116.30	115.40
O(10)-C(9)-C(8)	112.11	112.08	111.67	111.82	111.91
C(9)-C(8)-C(7)	110.74	110.19	110.34	109.46	110.36
C(8)-C(7)-C(6)	109.48	108.86	110.41	109.73	110.92
C(7)-C(6)-C(5)	124.65	124.20	124.71	124.24	123.81
C(6)-C(5)-C(4)	124.95	124.81	124.06	123.92	123.73
C(5)-C(4)-C(3)	110.86	109.96	111.57	110.77	112.41
C(4)-C(3)-C(2)	110.78	110.16	110.19	109.55	109.04
C(3)-C(2)-O(1)	112.25	112.55	111.84	112.07	112.43
**Bond Lengths (Å)**					
	A	B	C	D	XRD
O(1)-C(13)	1.46	1.44	1.46	1.44	1.45
C(13)-C(12)	1.52	1.52	1.53	1.52	1.51
C(12)-C(11)	1.51	1.51	1.52	1.52	1.50
C(11)-O(10)	1.45	1.43	1.45	1.43	1.44
O(10)-C(9)	1.35	1.34	1.35	1.34	1.34
C(9)-C(8)	1.50	1.50	1.52	1.51	1.49
C(8)-C(7)	1.54	1.53	1.55	1.53	1.53
C(7)-C(6)	1.50	1.50	1.51	1.51	1.50
C(6)-C(5)	1.33	1.33	1.34	1.33	1.31
C(5)-C(4)	1.50	1.49	1.51	1.50	1.49
C(4)-C(3)	1.53	1.53	1.54	1.54	1.52
C(3)-C(2)	1.51	1.50	1.52	1.51	1.50
C(2)-O(1)	1.35	1.34	1.35	1.34	1.33

A = B3LYP-D3/cc-PVDC
B = M06-2X-D3/cc-PVDC
C = B3LYP-D3/LACVP**
D = M06-2X-D3/LACVP**

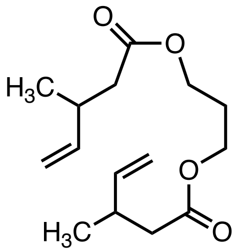

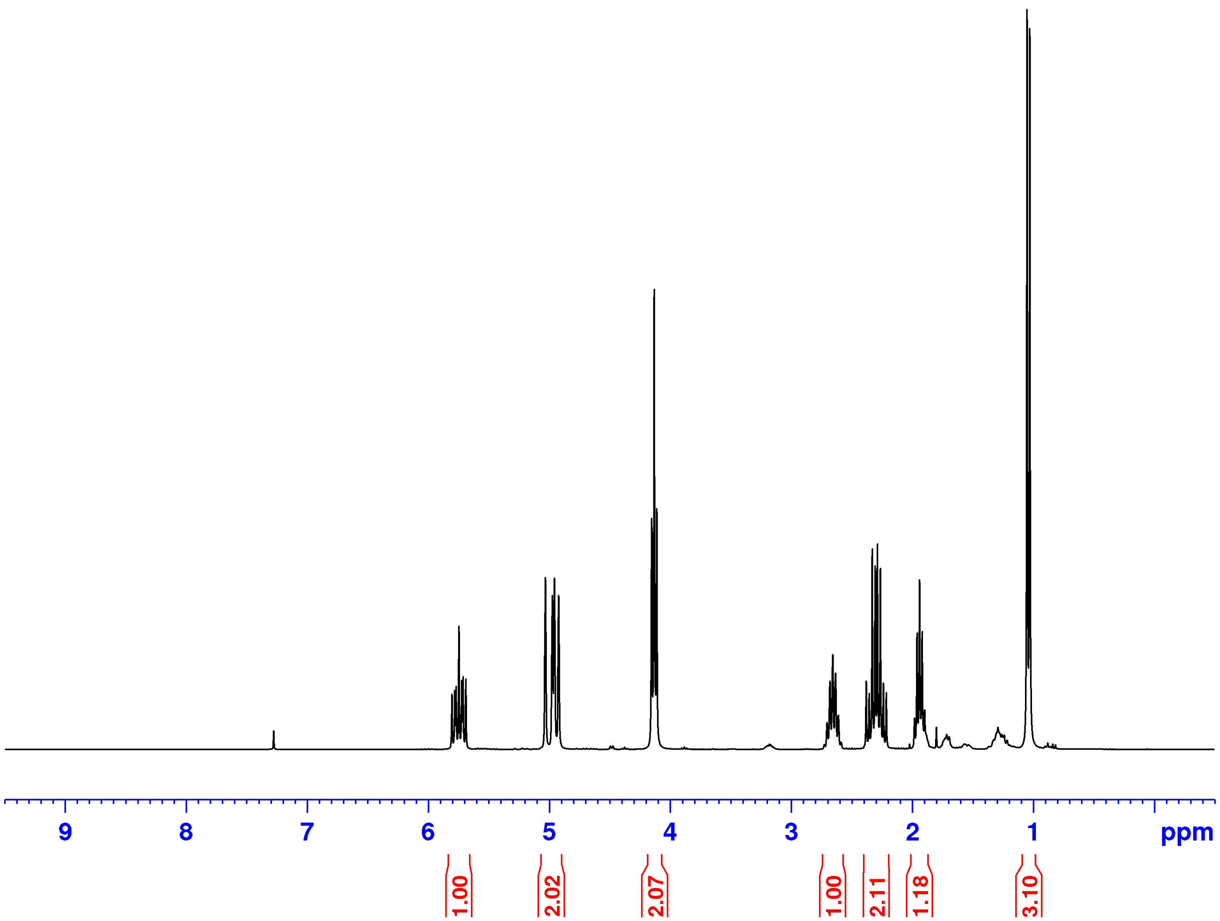


```
Current Data Parameters
NAME     kmr-mwp-34-spot7-9
EXPNO                     1
PROCNO                    1

F2 - Acquisition Parameters
Date_            20140110
Time                14.17
INSTRUM             spect
PROBHD   5 mm Multinucl
PULPROG              zg30
TD                  28900
SOLVENT             CDCl3
NS                     16
DS                      2
SWH              4873.294 Hz
FIDRES           0.168626 Hz
AQ              2.9651401 sec
RG                     57
DW                102.600 usec
DE                   6.50 usec
TE                  300.6 K
D1             2.00000000 sec
TD0                     1

======== CHANNEL f1 ========
NUC1                   1H
P1                   8.00 usec
PL1      0 dB
SFO1          300.1411075 MHz

F2 - Processing parameters
SI                  65536
SF            300.1400000 MHz
WDW                    EM
SSB      0
LB                   0.50 Hz
GB       0
PC                   1.00
```

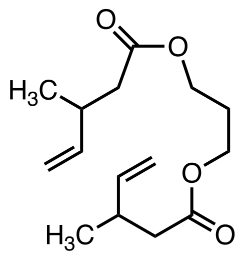

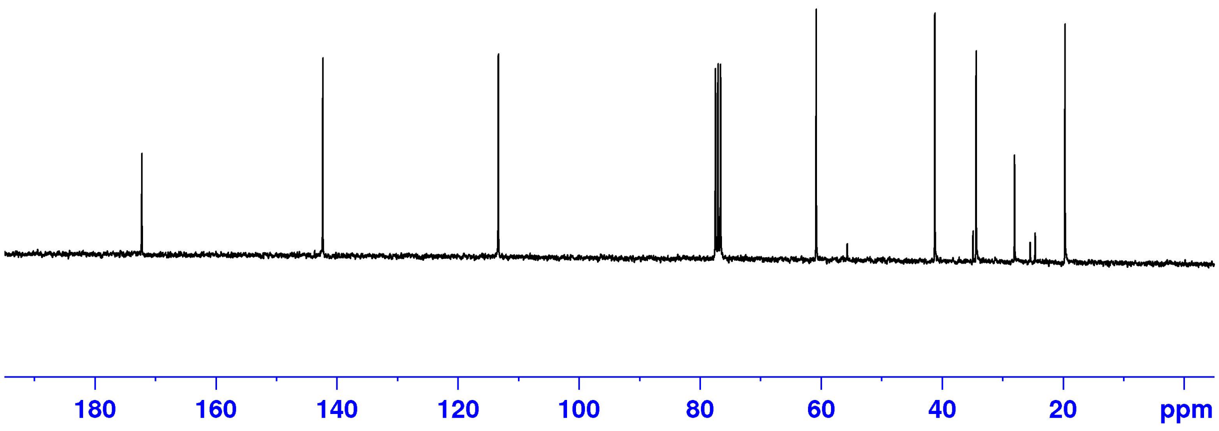


```
Current Data Parameters
NAME      kmr-mwp-34-spot7-9
EXPNO                      2
PROCNO                     1

F2 - Acquisition Parameters
Date_            20140110
Time                14.31
INSTRUM             spect
PROBHD   5 mm Multinucl
PULPROG           zgpg30
TD                 32696
SOLVENT            CDCl3
NS                   204
DS                     4
SWH            17985.611 Hz
FIDRES          0.550086 Hz
AQ             0.9089488 sec
RG                   181
DW                27.800 usec
DE                  6.50 usec
TE                 304.9 K
D1            1.50000000 sec
D11           0.03000000 sec
TD0                    1

======== CHANNEL f1 ========
NUC1                 13C
P1                  8.50 usec
PL1      0 dB
SFO1          75.4775590 MHz

======== CHANNEL f2 ========
CPDPRG[2         waltz16
NUC2                  1H
PCPD2             100.00 usec
PL2                -5.00 dB
PL12                8.56 dB
PL13               20.00 dB
SFO2         300.1402005 MHz

F2 - Processing parameters
SI                131072
SF            75.4702630 MHz
WDW                   EM
SSB      0
LB                  2.50 Hz
GB       0
PC                  1.40
```

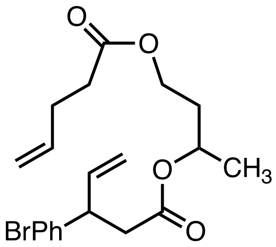

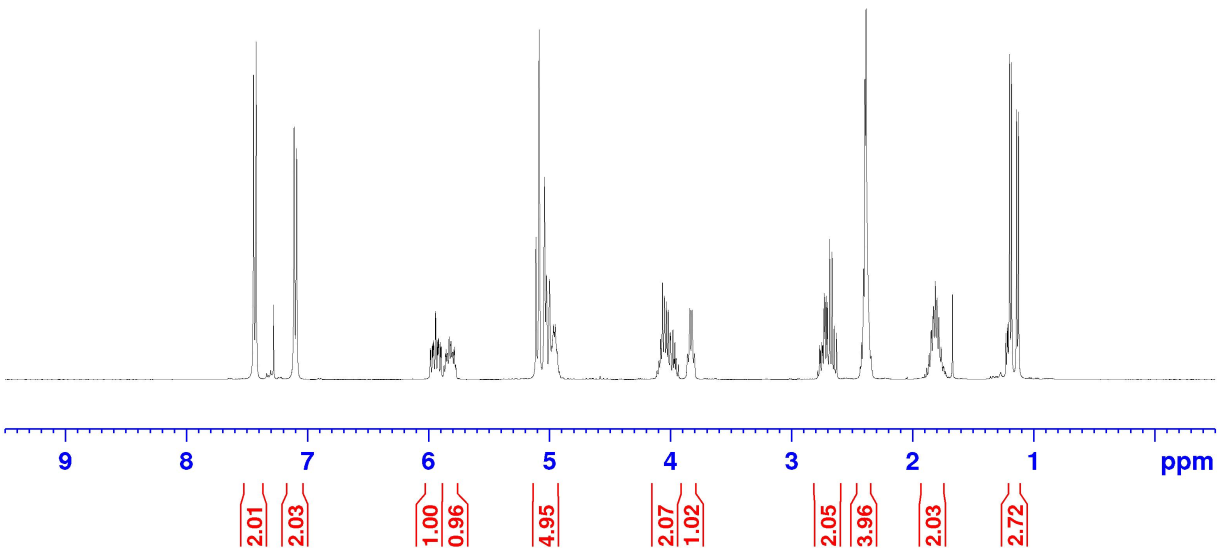


```
Current Data Parameters
NAME        kmr-mwp-43-c9
EXPNO                   1
PROCNO                  1

F2 - Acquisition Parameters
Date_            20140916
Time                15.27
INSTRUM             spect
PROBHD   5 mm PABBO BB/
PULPROG              zg30
TD                  48076
SOLVENT             CDCl3
NS                     16
DS                      2
SWH              8012.820 Hz
FIDRES           0.166670 Hz
AQ              2.9999423 sec
RG                   45.2
DW                 62.400 usec
DE                   6.50 usec
TE                  300.0 K
D1             2.00000000 sec
TD0                     1

======== CHANNEL f1 ========
NUC1                   1H
P1                  15.25 usec
PLW1          12.00000000 W
SFO1          400.1464009 MHz

F2 - Processing parameters
SI                 131072
SF            400.1440000 MHz
WDW                    EM
SSB      0
LB                   0.50 Hz
GB       0
PC                   1.00
```

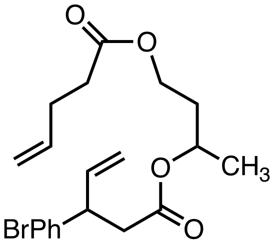

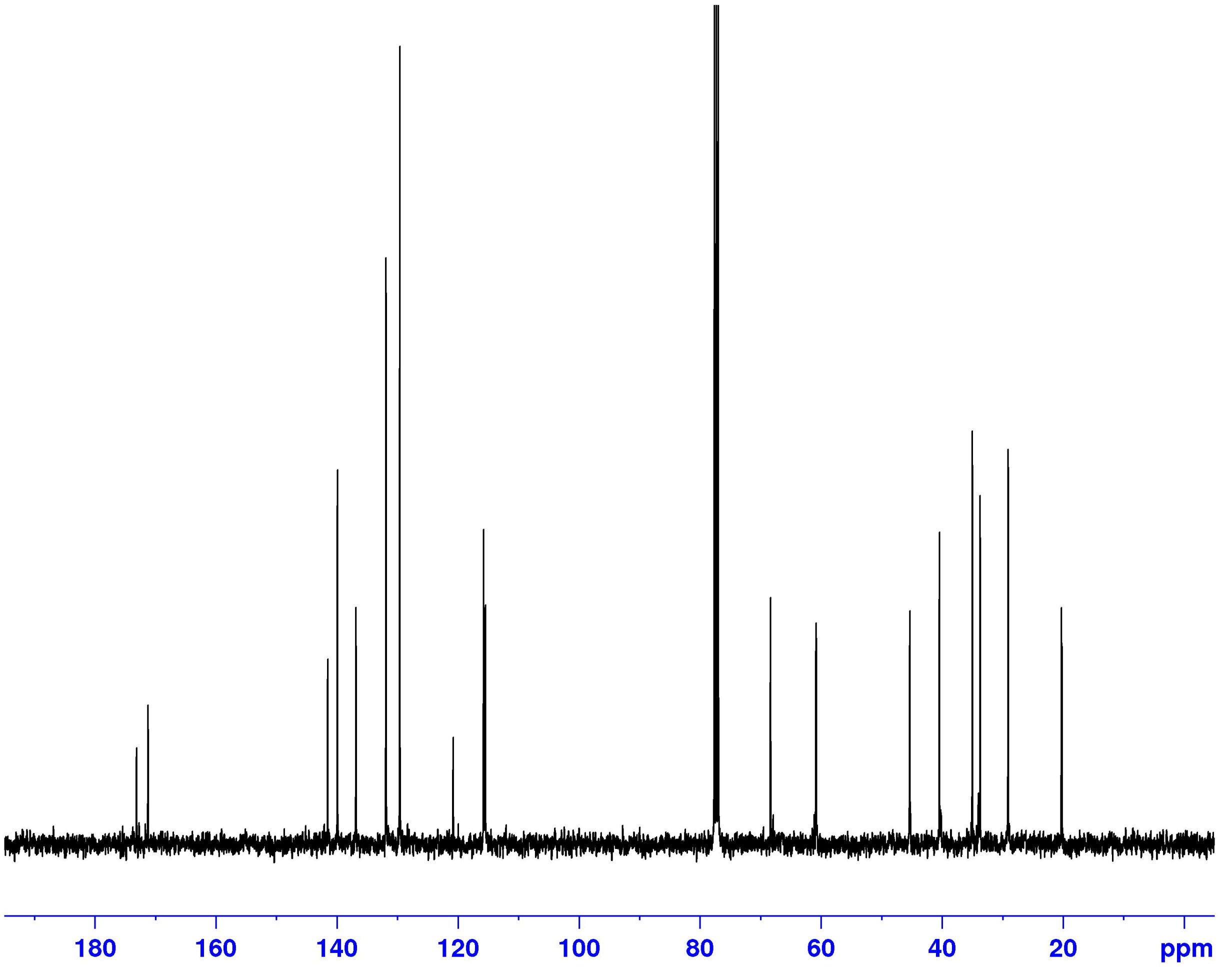


```
Current Data Parameters
NAME       kmr-mwp-43-c9
EXPNO                  2
PROCNO                 1

F2 - Acquisition Parameters
Date_          20140916
Time              15.37
INSTRUM           spect
PROBHD   5 mm PABBO BB/
PULPROG         zgpg30
TD               43698
SOLVENT          CDCl3
NS                 131
DS                   4
SWH          24038.461 Hz
FIDRES        0.550104 Hz
AQ           0.9089184 sec
RG                 203
DW              20.800 usec
DE                6.50 usec
TE               300.0 K
D1          1.50000000 sec
D11         0.03000000 sec
TD0                  1

======== CHANNEL f1 ========
NUC1               13C
P1               10.00 usec
PLW1       50.00299835 W
SFO1       100.6263500 MHz

======== CHANNEL f2 ========
CPDPRG[2        waltz16
NUC2                1H
PCPD2            90.00 usec
PLW2       12.00000000 W
PLW12       0.33333001 W
PLW13       0.27000001 W
SFO2       400.1456006 MHz

F2 - Processing parameters
SI              131072
SF         100.6162656 MHz
WDW                 EM
SSB      0
LB                2.50 Hz
GB       0
PC                1.40
```

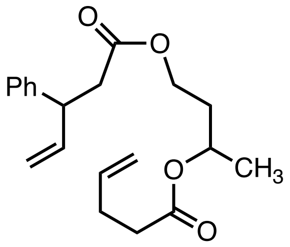

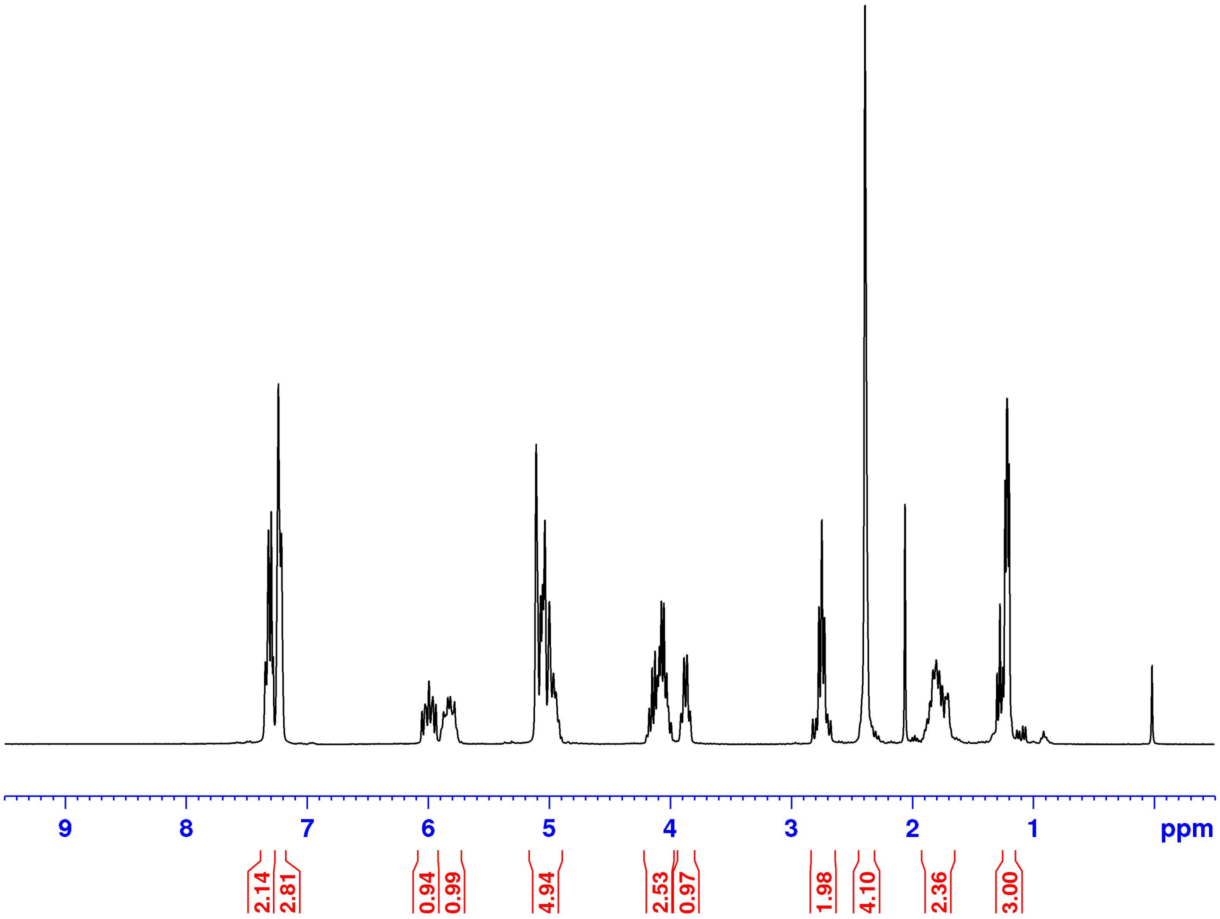


```
Current Data Parameters
NAME             kmr-mwp-55
EXPNO                     1
PROCNO                    1

F2 - Acquisition Parameters
Date_              20150610
Time                  16.57
INSTRUM               spect
PROBHD    5 mm Multinucl
PULPROG                zg30
TD                    28900
SOLVENT               CDCl3
NS                       16
DS                        2
SWH            4873.294 Hz
FIDRES         0.168626 Hz
AQ            2.9651401 sec
RG                     90.5
DW              102.600 usec
DE                 6.50 usec
TE                673.2 K
D1           2.00000000 sec
TD0                       1

======== CHANNEL f1 ========
NUC1                     1H
P1                 9.00 usec
PL1       0 dB
SFO1        300.1411075 MHz

F2 - Processing parameters
SI                    65536
SF          300.1400000 MHz
WDW                      EM
SSB       0
LB                  0.50 Hz
GB        0
PC                     1.00
```

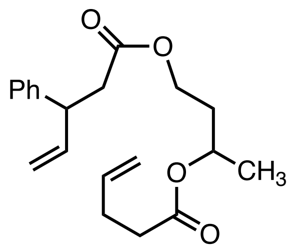

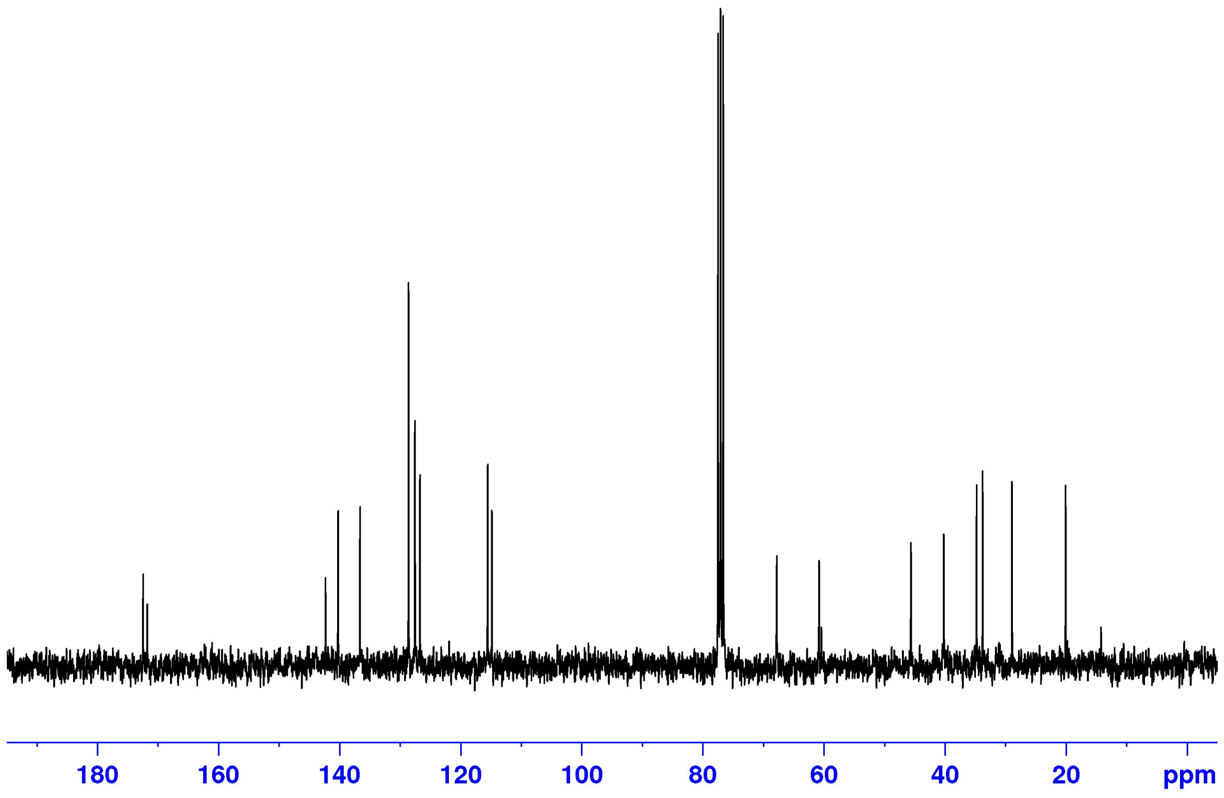


```
Current Data Parameters
NAME          kmr-mwp-55
EXPNO                  2
PROCNO                 1

F2 - Acquisition Parameters
Date_           20150610
Time               17.03
INSTRUM            spect
PROBHD   5 mm Multinucl
PULPROG          zgpg30
TD                 32696
SOLVENT            CDCl3
NS                   140
DS                     4
SWH            17985.611 Hz
FIDRES          0.550086 Hz
AQ             0.9089488 sec
RG                   181
DW                27.800 usec
DE                  6.50 usec
TE                 673.2 K
D1            1.50000000 sec
D11           0.03000000 sec
TD0                    1

======== CHANNEL f1 ========
NUC1                 13C
P1                 10.50 usec
PL1        0 dB
SFO1          75.4775590 MHz

======== CHANNEL f2 ========
CPDPRG[2         waltz16
NUC2                  1H
PCPD2             100.00 usec
PL2        0 dB
PL12               20.92 dB
PL13               20.00 dB
SFO2         300.1402005 MHz

F2 - Processing parameters
SI                131072
SF            75.4702630 MHz
WDW                   EM
SSB        0
LB                  2.50 Hz
GB         0
PC                  1.40
```

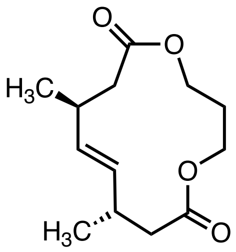

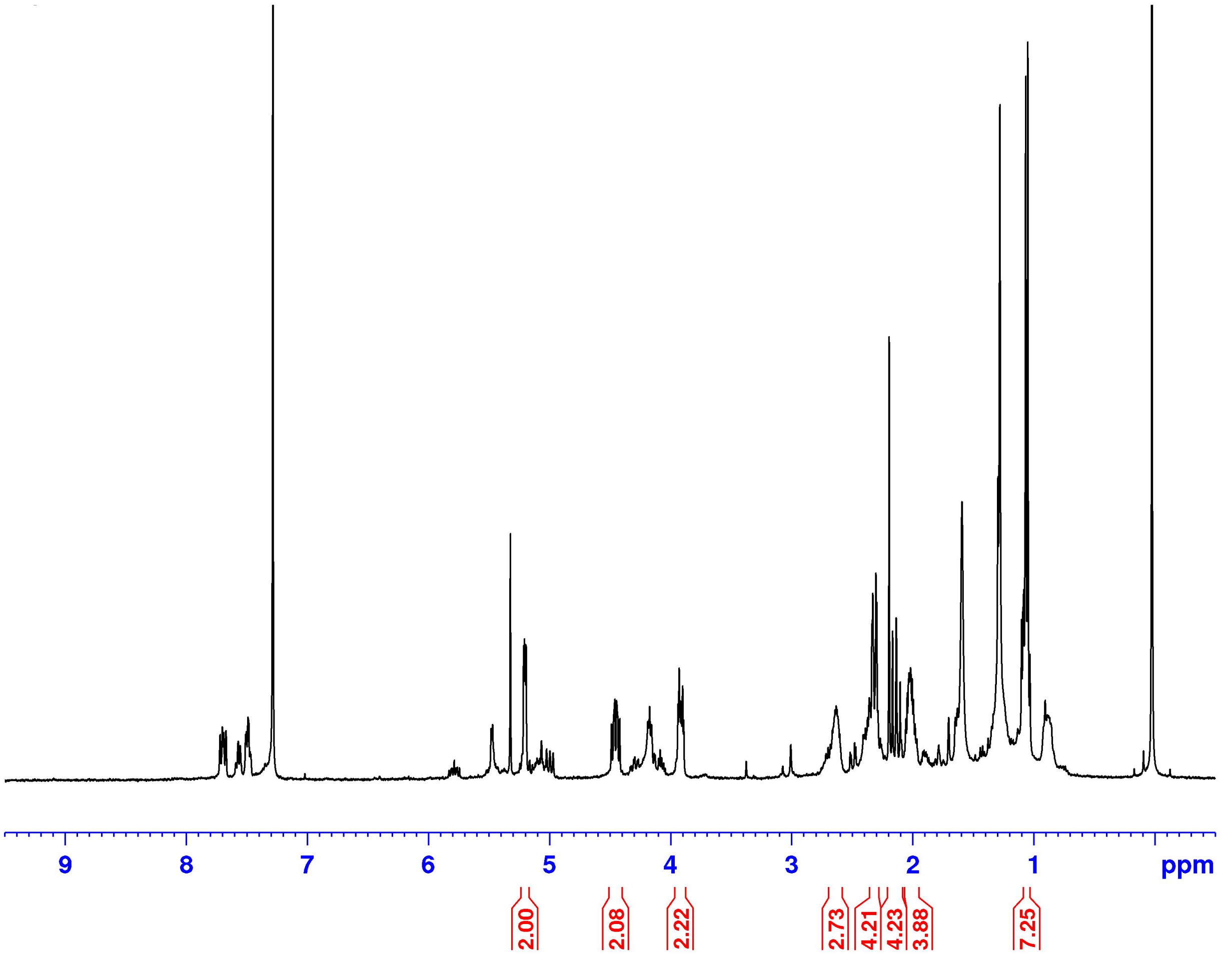


```
Current Data Parameters
NAME       kmr-mwp-60-spot-39-44
EXPNO                      1
PROCNO                     1

F2 - Acquisition Parameters
Date_             20150708
Time                 14.25
INSTRUM              spect
PROBHD    5 mm PABBO BB/
PULPROG               zg30
TD                   48076
SOLVENT              CDCl3
NS                      16
DS                       2
SWH               8012.820 Hz
FIDRES            0.166670 Hz
AQ               2.9999423 sec
RG                     203
DW                  62.400 usec
DE                    6.50 usec
TE                   300.0 K
D1              2.00000000 sec
TD0                      1

======== CHANNEL f1 ========
SFO1           400.1464009 MHz
NUC1                    1H
P1                   15.25 usec
PLW1           12.00000000 W

F2 - Processing parameters
SI                  131072
SF             400.1440000 MHz
WDW                     EM
SSB       0
LB                    0.50 Hz
GB        0
PC                    1.00
```

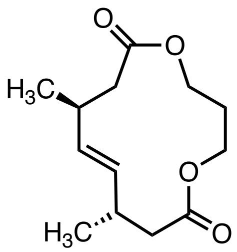

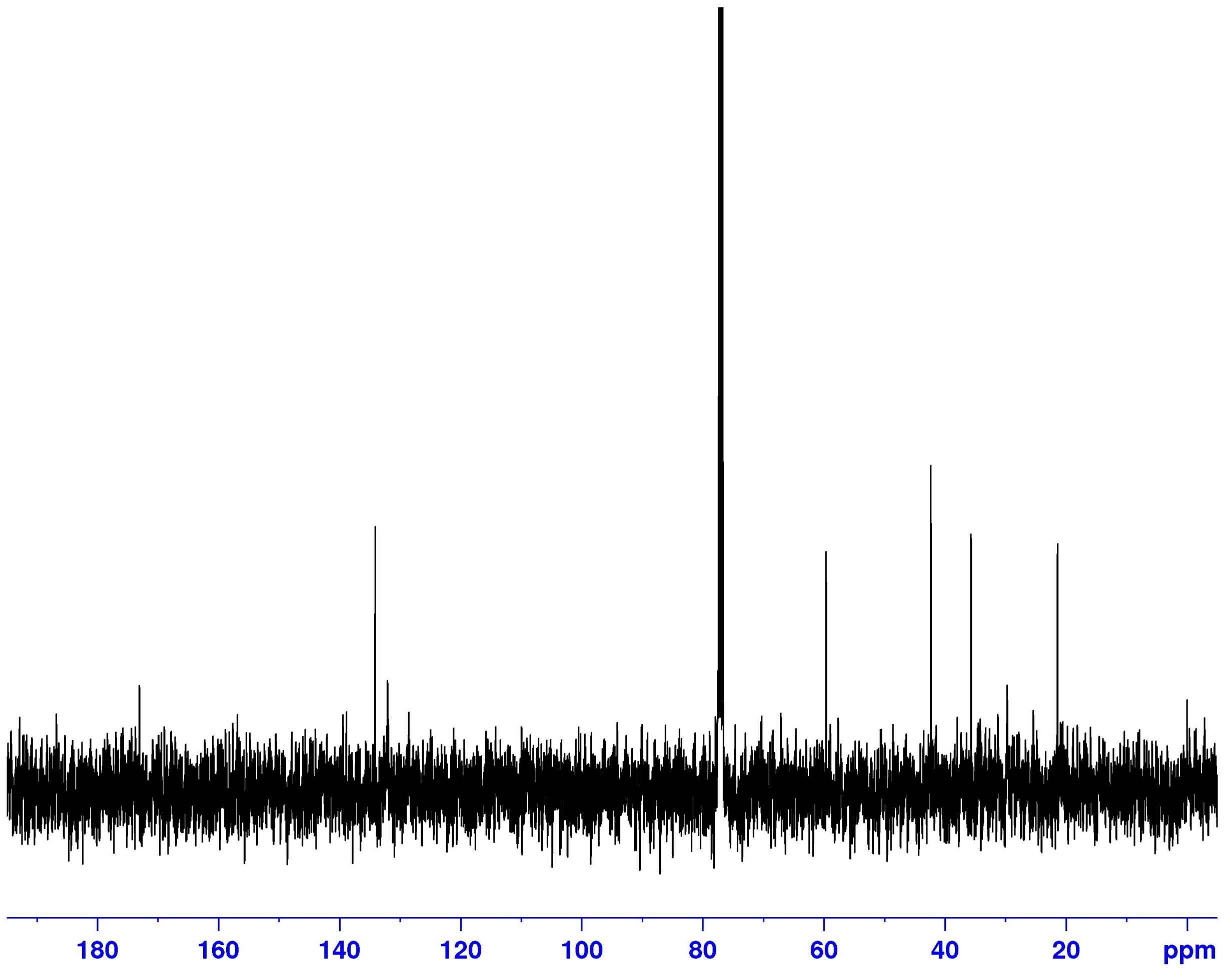


```
Current Data Parameters
NAME     kmr-mwp-60-spot-39-44
EXPNO                    2
PROCNO                   1

F2 - Acquisition Parameters
Date_           20150708
Time               14.41
INSTRUM            spect
PROBHD   5 mm PABBO BB/
PULPROG            udeft
TD                 17308
SOLVENT            CDCl3
NS                   151
DS                     0
SWH            24038.461 Hz
FIDRES          1.388864 Hz
AQ             0.3600064 sec
RG                   203
DW                20.800 usec
DE                  6.50 usec
TE                 300.0 K
D1            4.00000000 sec
D11           0.03000000 sec
D12           0.00002000 sec
D20         200.00000000 sec
TD0                    1

======== CHANNEL f1 ========
SFO1         100.6273568 MHz
NUC1                 13C
P1                 10.00 usec
P13              2000.00 usec
P26               500.00 usec
PLW1         50.00299835 W
SPNAM[5]    Crp60comp.4
SPOAL5             0.500
SPOFFS5  0 Hz
SPW5          7.63990021 W
SPNAM[8] Crp60,0.5,20.1
SPOAL8             0.500
SPOFFS8  0 Hz
SPW8          7.63990021 W

======== CHANNEL f2 ========
SFO2         400.1456006 MHz
NUC2                  1H
CPDPRG[2         waltz16
PCPD2              90.00 usec
PLW2         12.00000000 W
PLW12         0.33333001 W

F2 - Processing parameters
SI                131072
SF           100.6162890 MHz
WDW                   EM
SSB      0
LB                  2.00 Hz
GB       0
PC                  1.40
```

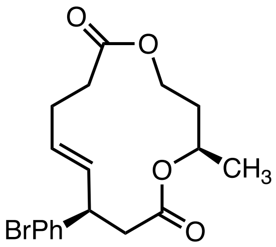

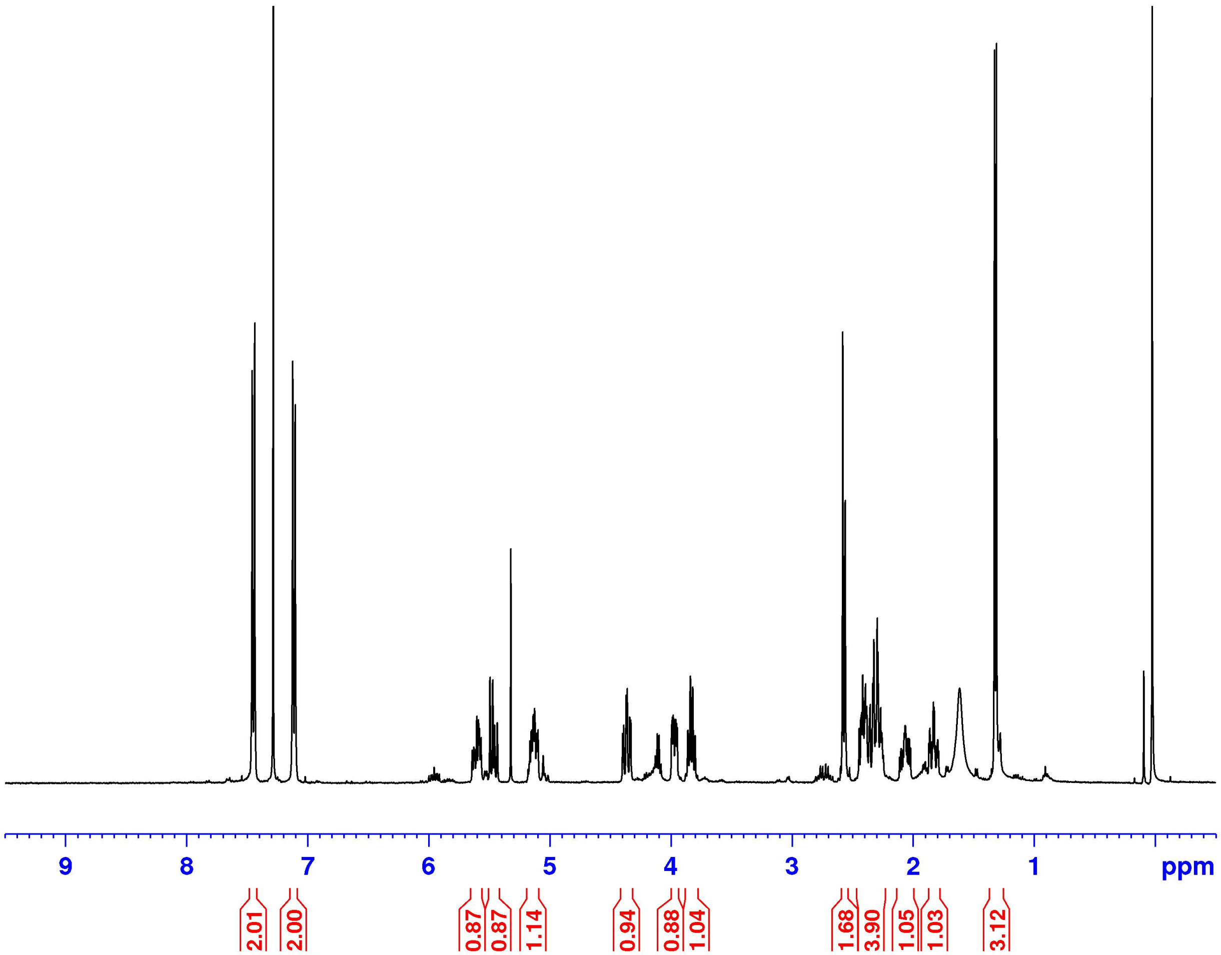


```
Current Data Parameters
NAME     kmr-mwp-65-spot13-18
EXPNO                       1
PROCNO                      1

F2 - Acquisition Parameters
Date_          20150817
Time              14.45
INSTRUM           spect
PROBHD   5 mm PABBO BB/
PULPROG            zg30
TD                48076
SOLVENT           CDCl3
NS                   16
DS                    2
SWH            8012.820 Hz
FIDRES         0.166670 Hz
AQ            2.9999423 sec
RG                  203
DW               62.400 usec
DE                 6.50 usec
TE                300.0 K
D1           2.00000000 sec
TD0                   1

======== CHANNEL f1 ========
SFO1        400.1464009 MHz
NUC1                 1H
P1                15.25 usec
PLW1        12.00000000 W

F2 - Processing parameters
SI               131072
SF          400.1440000 MHz
WDW                  EM
SSB      0
LB                 0.50 Hz
GB       0
PC                 1.00
```

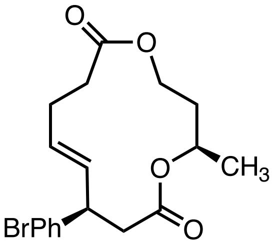

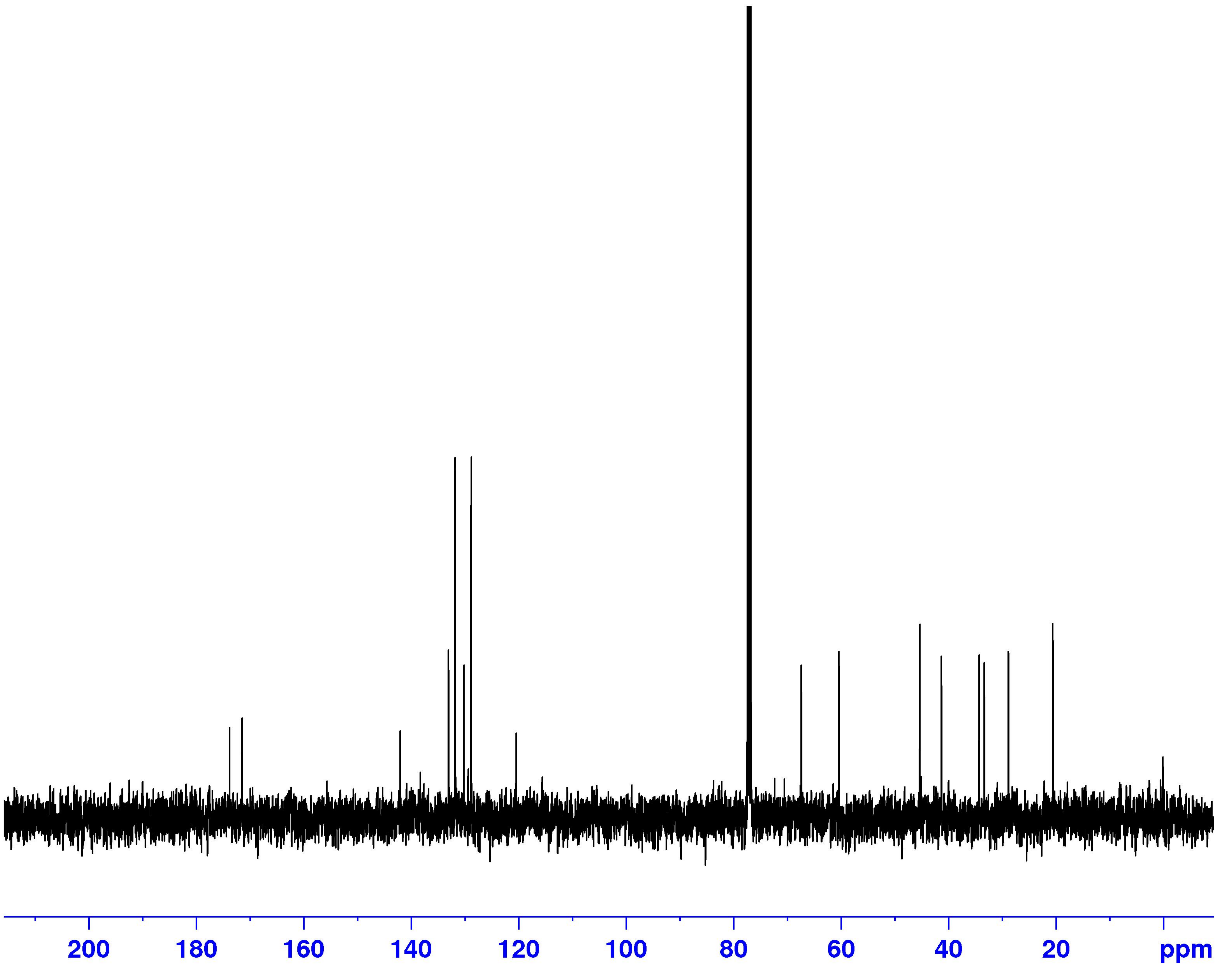


```
Current Data Parameters
NAME     kmr-mwp-65-spot13-18
EXPNO                    2
PROCNO                   1

F2 - Acquisition Parameters
Date_           20150817
Time               14.58
INSTRUM            spect
PROBHD   5 mm PABBO BB/
PULPROG           udeft
TD                17308
SOLVENT           CDCl3
NS                   92
DS                    0
SWH           24038.461 Hz
FIDRES         1.388864 Hz
AQ            0.3600064 sec
RG                  203
DW               20.800 usec
DE                 6.50 usec
TE                300.0 K
D1           4.00000000 sec
D11          0.03000000 sec
D12          0.00002000 sec
D20        200.00000000 sec
TD0                   1

======== CHANNEL f1 ========
SFO1        100.6273568 MHz
NUC1                13C
P1                10.00 usec
P13             2000.00 usec
P26              500.00 usec
PLW1        50.00299835 W
SPNAM[5]    Crp60comp.4
SPOAL5            0.500
SPOFFS5  0 Hz
SPW5         7.63990021 W
SPNAM[8] Crp60,0.5,20.1
SPOAL8            0.500
SPOFFS8  0 Hz
SPW8         7.63990021 W

======== CHANNEL f2 ========
SFO2        400.1456006 MHz
NUC2                 1H
CPDPRG[2        waltz16
PCPD2             90.00 usec
PLW2        12.00000000 W
PLW12        0.33333001 W

F2 - Processing parameters
SI               131072
SF          100.6162890 MHz
WDW                  EM
SSB      0
LB                 2.00 Hz
GB       0
PC                 1.40
```

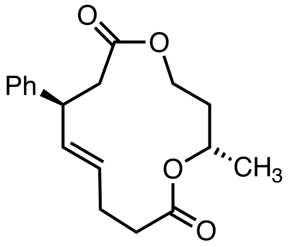

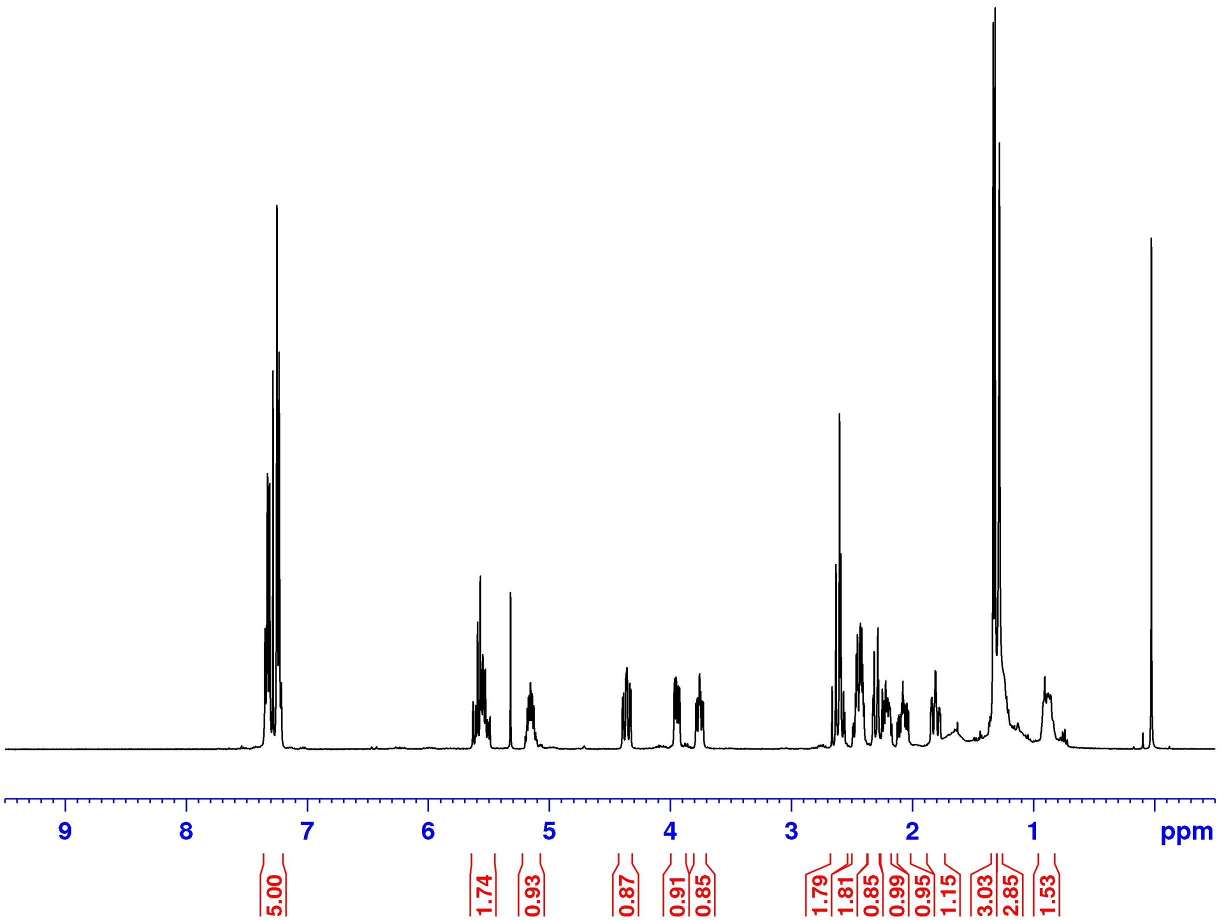

Current Data Parameters
NAME kmr-mwp-71-spot55-66
EXPNO 1
PROCNO 1

F2 - Acquisition Parameters
Date_ 20150708
Time 13.15
INSTRUM spect
PROBHD 5 mm PABBO BB/
PULPROG zg30
TD 48076
SOLVENT CDCl3
NS 16
DS 2
SWH 8012.820 Hz
FIDRES 0.166670 Hz
AQ 2.9999423 sec
RG 128
DW 62.400 usec
DE 6.50 usec
TE 300.0 K
D1 2.00000000 sec
TD0 1

======== CHANNEL f1 ========
SFO1 400.1464009 MHz
NUC1 1H
P1 15.25 usec
PLW1 12.00000000 W

F2 - Processing parameters
SI 131072
SF 400.1440000 MHz
WDW EM
SSB 0
LB 0.50 Hz
GB 0
PC 1.00

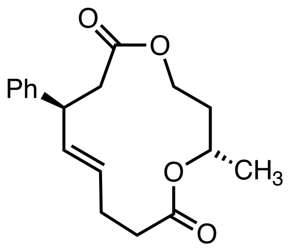

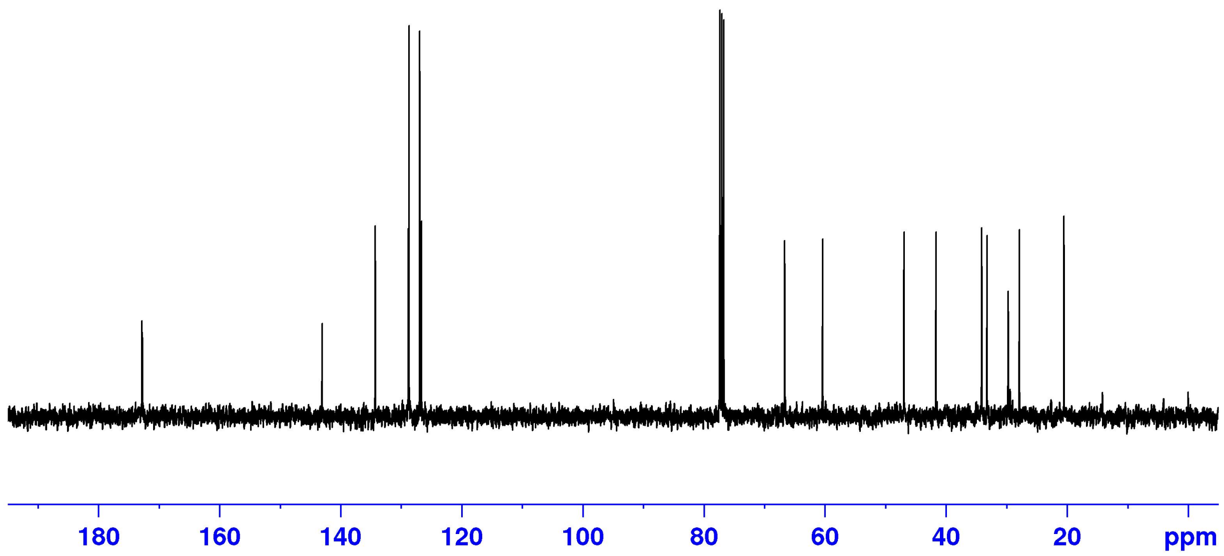


```
Current Data Parameters
NAME      kmr-mwp-71-spot55-66
EXPNO                    2
PROCNO                   1

F2 - Acquisition Parameters
Date_           20150708
Time               13.29
INSTRUM            spect
PROBHD   5 mm PABBO BB/
PULPROG            udeft
TD                 17308
SOLVENT            CDCl3
NS                   100
DS                     0
SWH            24038.461 Hz
FIDRES          1.388864 Hz
AQ             0.3600064 sec
RG                   203
DW                20.800 usec
DE                  6.50 usec
TE                 300.0 K
D1            4.00000000 sec
D11           0.03000000 sec
D12           0.00002000 sec
D20         200.00000000 sec
TD0                    1

======== CHANNEL f1 ========
SFO1         100.6273568 MHz
NUC1                 13C
P1                 10.00 usec
P13              2000.00 usec
P26               500.00 usec
PLW1         50.00299835 W
SPNAM[5]     Crp60comp.4
SPOAL5             0.500
SPOFFS5  0 Hz
SPW5          7.63990021 W
SPNAM[8] Crp60,0.5,20.1
SPOAL8             0.500
SPOFFS8  0 Hz
SPW8          7.63990021 W

======== CHANNEL f2 ========
SFO2         400.1456006 MHz
NUC2                  1H
CPDPRG[2         waltz16
PCPD2              90.00 usec
PLW2         12.00000000 W
PLW12         0.33333001 W

F2 - Processing parameters
SI                131072
SF           100.6162890 MHz
WDW                   EM
SSB      0
LB                  2.00 Hz
GB       0
PC                  1.40
```
